# Crucial roles of *Pox neuro* in the developing ellipsoid body and antennal lobes of the *Drosophila* brain

**DOI:** 10.1371/journal.pone.0176002

**Published:** 2017-04-25

**Authors:** Shilpi Minocha, Werner Boll, Markus Noll

**Affiliations:** Institute of Molecular Life Sciences, University of Zürich, Zürich, Switzerland; Biomedical Sciences Research Center Alexander Fleming, GREECE

## Abstract

The paired box gene *Pox neuro* (*Poxn*) is expressed in two bilaterally symmetric neuronal clusters of the developing adult *Drosophila* brain, a protocerebral dorsal cluster (DC) and a deutocerebral ventral cluster (VC). We show that all cells that express *Poxn* in the developing brain are postmitotic neurons. During embryogenesis, the DC and VC consist of only 20 and 12 neurons that express *Poxn*, designated embryonic *Poxn*-neurons. The number of *Poxn*-neurons increases only during the third larval instar, when the DC and VC increase dramatically to about 242 and 109 *Poxn*-neurons, respectively, virtually all of which survive to the adult stage, while no new *Poxn*-neurons are added during metamorphosis. Although the vast majority of *Poxn*-neurons express *Poxn* only during third instar, about half of them are born by the end of embryogenesis, as demonstrated by the absence of BrdU incorporation during larval stages. At late third instar, embryonic *Poxn*-neurons, which begin to express Poxn during embryogenesis, can be easily distinguished from embryonic-born and larval-born *Poxn*-neurons, which begin to express Poxn only during third instar, (i) by the absence of Pros, (ii) their overt differentiation of axons and neurites, and (iii) the strikingly larger diameter of their cell bodies still apparent in the adult brain. The embryonic *Poxn*-neurons are primary neurons that lay out the pioneering tracts for the secondary *Poxn*-neurons, which differentiate projections and axons that follow those of the primary neurons during metamorphosis. The DC and the VC participate only in two neuropils of the adult brain. The DC forms most, if not all, of the neurons that connect the bulb (lateral triangle) with the ellipsoid body, a prominent neuropil of the central complex, while the VC forms most of the ventral projection neurons of the antennal lobe, which connect it ipsilaterally to the lateral horn, bypassing the mushroom bodies. In addition, *Poxn*-neurons of the VC are ventral local interneurons of the antennal lobe. In the absence of Poxn protein in the developing brain, embryonic *Poxn*-neurons stall their projections and cannot find their proper target neuropils, the bulb and ellipsoid body in the case of the DC, or the antennal lobe and lateral horn in the case of the VC, whereby the absence of the ellipsoid body neuropil is particularly striking. *Poxn* is thus crucial for pathfinding both in the DC and VC. Additional implications of our results are discussed.

## Introduction

During metamorphosis, *Drosophila melanogaster* undergoes dramatic morphological changes, including extensive reorganization of the nervous system that transforms its control of larval behaviors into that of radically different and much more complex adult behaviors [1,2,3,4,5]. Many neurons are born during larval and pupal stages and become functional only in the nervous system of the adult fly. Other neurons that function only in the larva die during pupal development, while a third class of neurons, functional in the larva as well as the adult, exhibits pronounced remodeling of dendrites and axons during metamorphosis [4,6,7].

The central complex (CX) is part of the protocerebrum and the most prominent system of unpaired midline neuropils in the adult brain of hexapods and other arthropods [8,9,10,11,12,13]. Enclosed by a thin glial lamella, it is situated between the two supraesophageal brain hemispheres and composed of four inter-connected neuropils. In *Drosophila*, these are (from anterior to posterior) the ellipsoid body (EB), the fan-shaped body (FB) with the paired noduli below it, and the protocerebral bridge [10]. Neurons participating in these neuropils are classified as columnar small-field neurons and tangential large-field neurons, which form tracts perpendicular to those of the small-field neurons [10,14]. These interconnect small domains within a neuropil or between different neuropils of the CX, while large-field neurons typically arborize within an individual neuropil of the CX and extend their processes to other brain areas [10]. The large-field neurons of the toroid-like EB, also called ring or R neurons, form ring-shaped axonal projections in the EB and dendritic arborizations in the bulbs (formerly called lateral triangles) [10]. Enhancer trap lines specifically marking the neurons of the EB showed that this neuropil is formed by about 48 h APF (after puparium formation) at 25°C [14]. Anterior and ventrolateral to the CX, two glomerular neuropils of the antennal lobes (ALs) are located on either side of the esophagus [8,15,16,17,18]. In these neuropils, the tracts of the two antennal nerves deliver their information to the glomeruli of the ALs, from where it is passed on to other glomeruli of the ALs by local interneurons (LNs) and to higher brain centers, either directly to the lateral horn (LH) by ventral projection neurons (vPNs) or indirectly via the mushroom bodies (MB) to the LH by anterodorsal and lateral projection neurons, adPNs and lPNs [15,16,17,18].

A gene, previously shown to be expressed in the developing brain and in distinct cells of the adult brain that target the EB and ALs, is *Pox neuro* (*Poxn*) [19]. As a member of the Pax gene family, it encodes a transcription factor with a DNA-binding paired domain [20,21] and has many functions in various tissues, including the central and peripheral nervous system [19,20,22,23,24,25,26]. In the developing brain, Poxn protein is first detected in two bilaterally symmetric groups of cells of stage 12 embryos and continues to be expressed throughout development [19]. In the adult brain, Poxn is observed in a bilaterally symmetric dorsal and ventral cluster of neurons [19]. The major targets of the neurons in the dorsal cluster are the EB and the bulbs. The neurons of the ventral cluster target the AL [19] and LH. These may fall into two classes, so-called projection neurons, PNs, which connect the AL to higher brain centers like the MB and LH, and local interneurons, LNs, which connect the 50 glomeruli of the AL with each other [15,16,17].

All enhancers of *Poxn* and their functions had been identified previously [19] and allowed us to construct a *Poxn* transgene, *Poxn-Sbl* (S1 Fig), that expresses all *Poxn* functions except those required for transcription of *Poxn* in the developing and adult brain. Thus, by combining this transgene with the null mutant *Poxn*^*ΔM22-B5*^ [19], we were able to study the development of the brain structures, which express *Poxn* in the wild type, in the absence of Poxn without removing any *Poxn* functions expressed in the PNS or other parts of the developing fly.

By visualizing the neurites of neurons expressing *Poxn*, we have analyzed here (i) the precise timing of large-field neurons forming the EB, and (ii) the development of ventral projection neurons (vPNs) and ventral local interneurons (vLNs), which form part of the adult antennal lobe (AL). We demonstrate that most, if not all, of about 350 Poxn-expressing cells per brain hemisphere observed in late third instar larvae survive metamorphosis. Their projections follow the two paths laid down by the 20 and 12 embryonic Poxn-expressing cells of the DC and VC, respectively. These originate during embryogenesis and do not increase in number before the third larval instar. Several neurons of the DCs extend axons along tracts in the supraesophageal commissure (SEC) as early as stage 14 of embryogenesis that appear to meet at the midline and constitute the earliest trace of the adult EB neuropil. Massive elaboration of the axonal termini of these *Poxn*-neurons takes place within the EB, forming the complete toroid-like EB by 45 h APF. Neurites of the VC neurons, on the other hand, target the AL and LH in the larval, pupal, and adult brain. These neurons interconnect multiple glomeruli of the adult AL and connect directly, bypassing the MB, two salient adult olfactory centers, the AL and LH, through the mediolateral antennal lobe tract, mlALT [27], formerly called middle antennocerebral tract, mACT [15,18,28]. In the *Poxn* null mutant *Poxn*^*ΔM22-B5*^ [19], the projection pattern of *Poxn*-neurons is drastically disturbed. Axonal projections of the dorsal *Poxn*-neurons fail to form the structure of the EB, and the ventral *Poxn*-neurons no longer target the AL and LH. Our findings have many implications that are discussed.

## Materials and methods

### *Drosophila* strains and genetics

The following fly stocks and (re)combinations of their chromosomes were used:

*w*^*1118*^ (BL-5905),

*Ore-R* (Munich) (from W. McGinnis),

*P{UAS-FLP*.*Exel}1*, *y* *w*^*1118*^ (BL-8208),

*w*^*1118*^; *P{tubP-Gal80*^*ts*^*}20*; *TM2*/*TM6B*, *Tb*^*1*^ (BL-7019),

*P{Act5C>polyA>lacZ*.*nls1}3*, *ry*^*506*^ (BL-6355),

*w*^*1118*^; *P{W6 Poxn-CD8*::*GFP}3–3* (3rd chr.) [26],

*w*^*1118*^; *Poxn*^*ΔM22-B5*^/*CyO* [19],

*w*^*1118*^; *Poxn*^*ΔM22-B5*^ *P{W6 Poxn-Sbl}107*,

*w*^*1118*^; *Poxn*^*ΔM22-B5*^ *P{W6 Poxn-Sbl}44*,

*w*^*1118*^; *P{W6 Poxn-Gal4}13-1*/*TM6B* [19],

*w*^*1118*^; *P{W6 Poxn-Gal4}13–1 P{y*^+^ *UAS-GFP}/TM6B* [19],

*w*^*1118*^; *Poxn*^*ΔM22-B5*^ *P{W6 Poxn-SuperA}158* [25],

*w*^*1118*^; *P{UAS-CD2}5* (BL-1284),

*w*^*1118*^; *P{Gal4}repo/TM3*, *Sb*^*1*^ (BL-7415).

The *Poxn-CD8*::*GFP* transgene (S1 Fig) was constructed by combining three DNA fragments. The first was obtained as *Not*I–*Age*I fragment from the *Poxn* rescue construct *EvK* [19], which consisted of the *Poxn* coding region downstream of the *Not*I site in the last exon of *Poxn* (exon 5) followed by the 3’UTR, the pW6 vector of *EvK*, and the upstream control region of *Poxn* from *Hin*dIII (at -8202) to *Age*I (at -137 in S1 Fig). The second fragment was obtained as *Age*I–*Nco*I fragment from the *Poxn-Gal4*^*ups1f*^ construct [26], which consisted of 137 bp upstream region of *Poxn* followed by the 5’ UTR up to an artificial *Nco*I site including the translational start site of *Poxn* (S1 Fig). These fragments were combined with a PCR fragment, linked to a *Nco*I *Poxn*-primer and a *Not*I GFP-primer at its ends and encoding the mCD8::GFP fusion protein (S1 Fig). The *mCD8*::*GFP* DNA was that of the *mCD8-GFP/pUAST* transgene [29]. The DNA sequence of this *Poxn-CD8*::*GFP* transgene is available on request. It is under the direct control of the *Poxn* upstream region that includes the brain enhancer (S1 Fig), expresses the alpha chain of the mouse lymphocyte receptor CD8 fused to GFP, and was used to reveal neuronal processes [29]. It is also expressed in the Poxn domain of the developing and adult brain in the absence of *Poxn* function. Its expression in the brain coincides completely with that of Poxn protein in the wild type throughout development, but it exhibits ectopic expression in about 20 neurons closer to the midline than, and posterior to, the ventral clusters of Poxn expression in the adult. Ectopic expression of *Poxn-CD8*::*GFP* in late third instar larvae, however, appears negligible. The *Poxn-Sbl* transgene includes the entire *Poxn* gene except a 1.442 kb *Asc*I-*Eco*RI upstream fragment, the absence of which inactivates only the brain enhancer (S1 Fig).

### Dissection, immunolabeling, and microscopy of larval, pupal, and adult brains

Larval stages were determined by measuring the elapsed time after egg laying (AEL) of synchronized embryos at 25°C (see legend to Table 1) after embryos that had not hatched after 24 h had been discarded, while pupal stages were assessed as described [30]. Larval and pupal brains with ventral cords and adult brains were dissected in *Drosophila* Ringer’s solution (4.7 mM KCl, 130 mM NaCl, 2.0 mM CaCl_2_, 10 mM HEPES-NaOH, pH 6.9) with Dumont #55 (Dumostar) Tweezers. After dissection, the tissue was fixed for up to 2 h in 4% formaldehyde (Fluka 47629) in PEM buffer (0.1 M PIPES-NaOH, pH 7.0, 2 mM EGTA, 1 mM MgCl_2_, 0.02% NaN_3_) at 4°C, fixed for another 2 h at room temperature, rinsed once in PBS (130 mM NaCl, 7 mM Na_2_HPO_4_, 3 mM KH_2_PO_4_, pH 7.0), and permeabilized for 15 min at room temperature with 1% Triton X-100 (Fluka 93418) in PBS. After the brains had been rinsed twice in PBST (PBS supplemented with 0.2% Tween-20, Fluka 93773), they were blocked with 5% normal goat serum (NGS, Sigma G6767) in PBST for 60 min at room temperature, and incubated overnight at 4°C with appropriate dilutions of the preabsorbed primary antiserum in PBST, 3% NGS on a shaking platform. All primary and secondary antisera were preabsorbed before use in PBST, 3% NGS with 20% (v/v) fixed 0–4 h-old embryos at 4°C overnight. After several washes in PBST, appropriate dilutions of the preabsorbed secondary antiserum in PBST, 3% NGS were added and brains were incubated overnight at 4°C. Brains were then washed several times in PBST and equilibrated in mounting medium (80% Glycerol, 0.1% DABCO (1,4-Diazabicyclo[2.2.2]octane, Fluka 33480), 0.1 M Tris-HCl, pH 7.5). The brain whole-mounts were mounted with the cover slip supported by an appropriate spacer of about 150 μm for subsequent analysis by confocal microscopy. During mounting, the larval brain was rotated anteriorly, thus flattening the CNS and eliminating the typical 90° rotation of the CNS at the intersection between the brain and the ventral nerve cord, such that the A/P axis of the brain becomes the Z-axis in the confocal microscope [31]. In few cases, the larval brain was rotated posteriorly by 90°, like in S2B Fig, such that it was pressed down onto the ventral nerve cord.

**Table 1:** Number of Poxn-expressing cells per brain hemisphere in embryos, larvae, and adults.

Developmental stage^†^	Number of *Poxn*-neurons per brain hemisphere*			
	*Oregon-R*		*Poxn* mutant^¶^	
10.5–11.5 h AEL (st. 14)	16–22	(DC: 8–12; VC: 8–10)	18–22	(DC: 8–12; VC: 10)
12–13 h AEL (late st. 15)	28–30	(DC: 16–18; VC: 12)	30	(DC: 18; VC: 12)
15–16 h AEL (late st. 16/st. 17)	30–32	(DC: 18–20; VC: 12)	28–30	(DC: 16–18; VC: 12)
end of first larval instar (45 h AEL)	32	(DC: 20; VC: 12)	32	(DC: 20; VC: 12)
end of second larval instar (68 h AEL)	32	(DC: 20; VC: 12)		
third larval instar (wandering stage)	350	(DC: 242; VC: 108)		
adult	351	(DC: 242 ± 6; VC: 109 ± 2)		

*Numbers in adult brains and their standard deviations were determined by counting the neurons expressing Poxn protein in four clusters each of the DC and VC (see Materials and methods). The numbers of *Poxn*-neurons in the DC (20) and VC (12) appear to remain constant during late embryogenesis and the first two larval instars (S2 Fig), as determined for at least two clusters each in embryonic brains and first and second instar larval brains. Similarly, it is possible that the numbers of *Poxn*-neurons in the DC and VC of the adult brain do not vary between different brains and that the standard deviations only reflect the difficulty to distinguish in very few cases, similar to the standard deviations, whether the nuclear Poxn signal derives from one or two closely spaced nuclei in the confocal image. The number of *Poxn*-neurons in late third instar brains was counted in an adult brain, the *Poxn*-neurons of which had been labeled permanently by the expression of nuclear β-Gal (see Materials and methods). The numbers of *Poxn*-neurons in *Poxn* mutant third instar and adult brains are probably similar to those in the corresponding wild-type brains (cf. Materials and methods). DC, protocerebral dorsal cluster; VC, deutocerebral ventral cluster.

^†^Embryos were staged, after two 1-hour precollections and a 1-hour collection at 25°C, according to morphological markers or time of development at 25°C. The times after egg laying (AEL) do not take into account the time period between collection of embryos and their fixation for analysis, which was about 20 minutes. Larvae were staged according to time of development at 25°C and morphology (mouthhooks). Adults were 5 days old.

^¶^Genotype: *Poxn*^*ΔM22-B5*^; *Poxn-CD8*::*GFP*

The following primary and secondary antibodies were used: rabbit anti-Poxn (at a 1:50 dilution [20]), chicken anti-GFP (1:500; Abcam, Cambridge, UK), rabbit anti-β-galactosidase (1:100; Upstate Biotechnology, Lake Placid, NY), mouse anti-β-galactosidase (1:500; Promega), mouse anti-CD2 (1:100; Santa Cruz Biotechnology), mouse anti-Elav-9F8A9 (1:200), mouse 1D4 anti-Fasciclin II (1:2.5), mouse 4F3 anti-Discs Large (1:100), mouse anti-Prospero MR1A (1:4), mouse nc82 (1:10), mouse anti-BrdU (G3G4) (1:200), mouse anti-8D12 (anti-Repo) (1:200) (all these monoclonal antibodies are from Developmental Studies Hybridoma Bank, University of Iowa), Alexa Fluor 488-coupled goat anti-mouse IgG, Alexa Fluor 488-coupled goat anti-rabbit IgG, Alexa Fluor 488-coupled goat anti-chicken IgY, Alexa Fluor 594-coupled goat anti-mouse IgG, and Alexa Fluor 594-coupled goat anti-rabbit IgG (all secondary antibodies are from Molecular Probes and were used at a 1:500 dilution).

Labeled brains were analyzed with a Leica TCS SP or a Zeiss LSM 710 confocal laser scanning microscope (CLSM). Optical sections ranged from 0.50 μm to 1.0 μm, and signals of different fluorochromes were recorded sequentially with a resolution of 1024x1024 pixels. The resulting Z-stacks were arranged and processed by use of Leica ‘LCS lite’ or ‘Zeiss Zen 2009 light edition’ and the NIH ImageJ software version 1.41m (http://rsb.info.nih.gov/ij/) [32,33,34,35]. Z-stacks did not extend over the full thickness of the brains but rather were chosen to include all *Poxn*-neurons and occasionally extended beyond these limits, which is why the dimensions of entire Z-stacks, indicated in the legends to the figures, may not always reflect relevant dimensions of the brains. Analysis of colocalization was performed in ImageJ with the plugins Colocalization Threshold and Colocalization Highlighter [36], and by visual inspection of individual layers with the Imaris software.

### Analysis of mitotic activity in *Poxn*-neurons by incorporation of BrdU

BrdU (5-bromo-2-deoxyuridine; Sigma, B9285), dissolved in 40% ethanol at 10 mg/ml, was mixed with fly food at a concentration of 0.2 mg/ml [37]. Eggs were transferred to this BrdU-containing food such that larvae were labeled by BrdU from the time of hatching till wandering third instar. Feeding was monitored after a day or occasionally after a few hours by the uptake of the vital dye phenol red, added in trace amounts to the food, and non-feeding larvae were discarded. Adult brains were dissected in *Drosophila* Ringer’s solution and immediately fixed in 4% formaldehyde, PEM buffer at 4°C for 2 h. After fixation, the brains were washed 3 times for 15 min each in PBST at room temperature, incubated in 2 M HCl for 45 min to denature the BrdU-labeled DNA, again washed 3 times in PBST for 15 min each, and immunostained as described above.

### Permanent labeling of larval *Poxn*-neurons

Permanent labeling of larval *Poxn*-neurons was achieved by Flippase-mediated recombination, which was controlled in space by the Gal4/UAS system and in time by heat inactivation of the temperature-sensitive Gal80^ts^ repressor [38,39]. *w*^*1118*^ *UAS-Flp*/(+ or *Y*); *tub-Gal80*^*ts*^/+; *Act5C>polyA>lacZ*.*nls1 Poxn-Gal4-13-1*/*Poxn-CD8*::*GFP* third instar larvae, grown at 18°C and 65% relative humidity, were heat-shocked during their feeding stage for 6 hours by transfer to a prewarmed culture tube in a 32°C waterbath, and allowed to develop to adulthood by returning the tube to 18°C. *Poxn*-neurons of third instar larvae, in which flip-out has occurred, express nuclear β-Gal under the control of the constitutive actin promoter, while the *Poxn-CD8*::*GFP* transgene labels all *Poxn*-neurons throughout brain development. Eggs were collected from *w*^*1118*^ *UAS-Flp*/+; *tub-Gal80*^*ts*^; *Poxn-CD8*::*GFP* virgins crossed with *Act5C>polyA>lacZ*.*nls1 Poxn-Gal4-13-1*/*TM6B* males over an extended period of up to 12 hours to obtain a sufficiently large number of third instar larvae of which, after heat shock (at 6.5 days AEL), only very few survived to adulthood. In the three adult brains examined, virtually all *Poxn*-neurons also expressed β-Gal. It thus appears that these brains had all been heat-shocked during late feeding stage of third instar larvae (Table 1).

No leakiness of Gal80^ts^ was observed at 18°C, as demonstrated in control experiments in which *w*^*1118*^; *tub-Gal80*^*ts*^; *TM2/TM6B*, *Tb*^*1*^ virgins were crossed with *w*^*1118*^*/Y*; *Poxn-Gal4-13-1 UAS-GFP/TM6B* males. The *Tb*^+^ offspring larvae with the genotype *w*^*1118*^*/w*^*1118*^ or *Y*; *tub-Gal80*^*ts*^/+; *Poxn-Gal4-13-1 UAS-GFP/TM2* were grown at 18°C. They were heat-shocked at 30°C or 32°C for 6 hours during their feeding stage by transfer to a prewarmed culture tube. Some larvae were then returned to 18°C and allowed to develop to the late third instar or adult stage. No leaky expression of *UAS-GFP* under control of *Poxn-Gal4-13-*1 was observed in these late third instar or adult brains at 18°C whereas larvae, which continued to develop at 30°C to the late third instar, showed *Poxn*-specific expression of GFP in the brain.

### Cell counts of *Poxn*-neurons in embryonic, larval, and adult brains

The number of *Poxn*-neurons was determined by numbering the labeled *Poxn*-nuclei in each layer of the confocal image, starting from one end, and assigning to the same nucleus visible in adjacent layers the same number and different nuclei increasing numbers. This is a very tedious task, but results in much more reliable cell counts than any of the programs like ImageJ, which critically depend on threshold values that are impossible to set reliably because most nuclei of *Poxn*-neurons are closely clustered in the DC and VC. Numbers of *Poxn*-neurons in *Poxn* mutant brains of third instar larvae and adults are probably similar to those in the wild-type brains but could not be determined reliably because staining of *Poxn*-neurons with anti-GFP for membrane-bound GFP in mutants is less sensitive than that of nuclei labeled by anti-Poxn in wild-type brains. This is in analogy to the POU domain transcription factor Acj6, which is expressed in every adPN but not in vPNs [18], which express Poxn. Also in this case, no significant change in cell numbers of mutant compared to wild-type adNB clones has been observed in adult brains [40].

## Results

### Expression of Poxn in brain cells begins during embryogenesis and third larval instar

In the brain, Poxn protein initially appears bilaterally at stage 12 of embryogenesis [19] in a single cell of the deutocerebrum [41]. By stage 13, it is expressed in small clusters of the protocerebrum and deutocerebrum [41,42] where it continues to be expressed with bilateral symmetry in a protocerebral and a deutocerebral cluster throughout development [19]. By stage 14, the protocerebral cluster is dorsal and, with regard to the body axis, slightly posterior to the deutocerebral cluster, yet anterior with regard to the axis of the CNS [42]. To avoid confusion, we therefore refer to the protocerebral cluster as dorsal cluster (DC) and the deutocerebral cluster as ventral cluster (VC). The number of cells in the VC and DC reaches 12 and 20 by stage 15 and 17 of embryogenesis, respectively (Table 1). These numbers remain constant during the first and second larval instars (Table 1; S2 Fig). Beginning with the third instar, however, the number of Poxn-expressing cells per brain hemisphere increases dramatically, reaching about 242 and 109 cells in the DC and VC of adult brains, respectively (Table 1).

**Fig 1:**
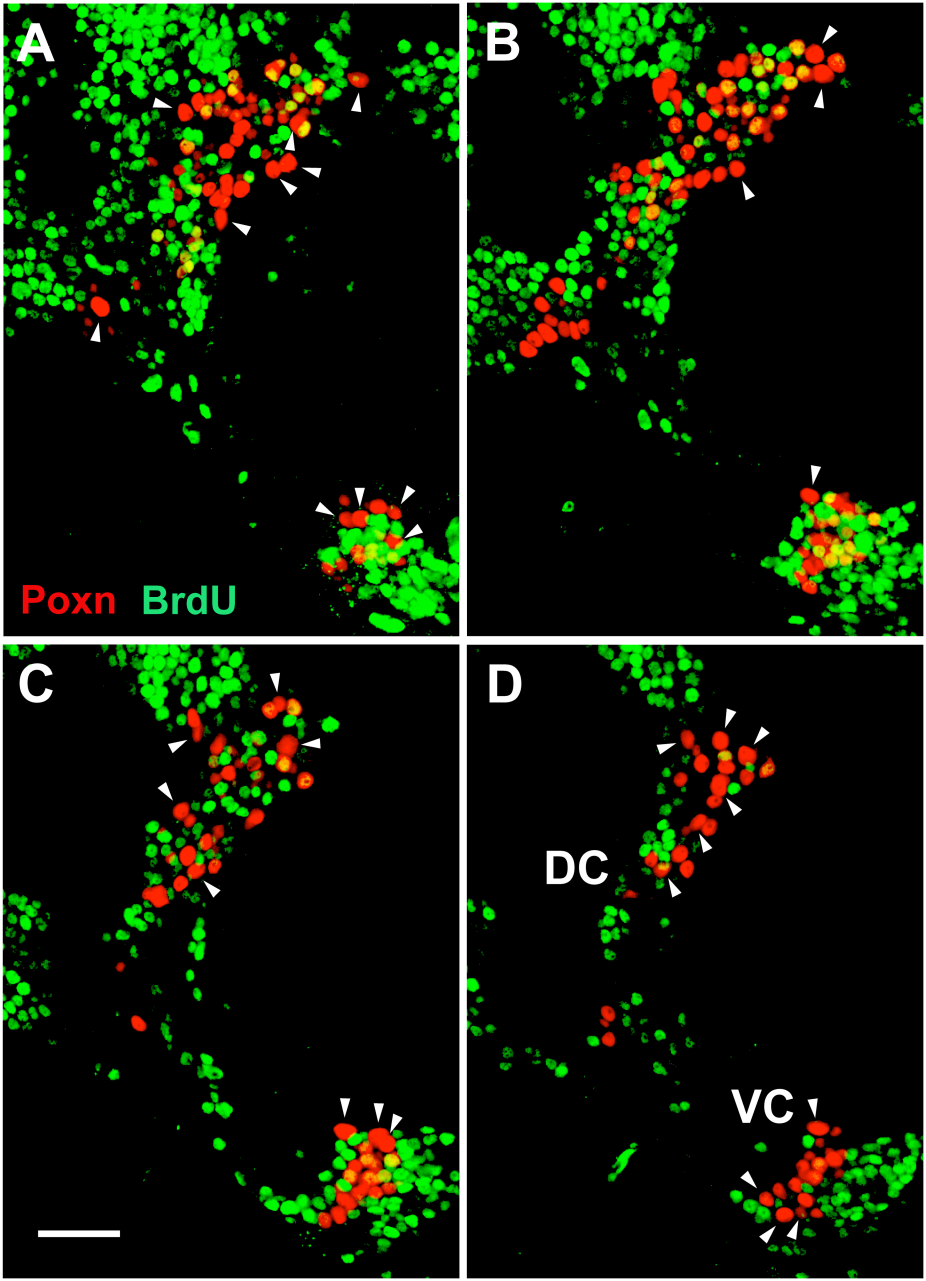
At least half of the Poxn-expressing cells in the adult brain completed their last S-phase during embryogenesis. BrdU fed throughout larval development and incorporated into DNA was analyzed in an adult *Ore-R* brain in CLSM sections at 63x magnification. *Poxn*-neurons of one brain hemisphere, stained for Poxn (red) and incorporated BrdU (green), are shown in 1 μm sections at 4–5 μm (A), 7–8 μm (B), 10–11 μm (C), and 13–14 μm (D) of a Z-stack extending from 0 (anterior) to 30 μm (posterior). Colocalization of BrdU with Poxn was analyzed in each Poxn-labeled nucleus by careful inspection of single confocal layers. Arrowheads point at the 20 and 12 largest *Poxn*-nuclei of the DC and VC, respectively, all of which are free of BrdU, and thus are thought to correspond to the 20 (DC) and 12 (VC) *Poxn*-neurons that express Poxn during embryogenesis and lack Pros in late third instar larval brains (S5, S7A and S7B Figs, Table 1). Note that some of these *Poxn*-nuclei appear smaller than other BrdU-negative *Poxn*-nuclei in the same section but are actually larger, as evident from adjacent sections (not shown). Scale bar: 20 μm.

Thus, most adult brain cells that express Poxn begin to express it only during the third instar, a criterion by which they can be distinguished from the 32 cells that already express Poxn during embryogenesis. To test whether the large majority of cells that initiate Poxn expression after embryogenesis are also born during larval stages, incorporation of BrdU fed throughout larval development was analyzed in adult brains. Surprisingly, only 48% of the cells of the DC and 42% of the cells of the VC incorporated BrdU, as evident from careful inspection of single confocal layers of a Z-stack (Fig 1). By contrast, all but 32 cells that express Poxn in the adult brain (20 in the DC and 12 in the VC; Table 1) begin to express Poxn only during third instar, i.e., 92% in the DC and 89% in the VC. In other words, in addition to the 32 cells that express Poxn by the end of embryogenesis, almost half of the cells that initiate Poxn expression during third instar have completed their last S-phase during embryogenesis.

### Cells that express Poxn in the developing brain are post-mitotic neurons

**Fig 2:**
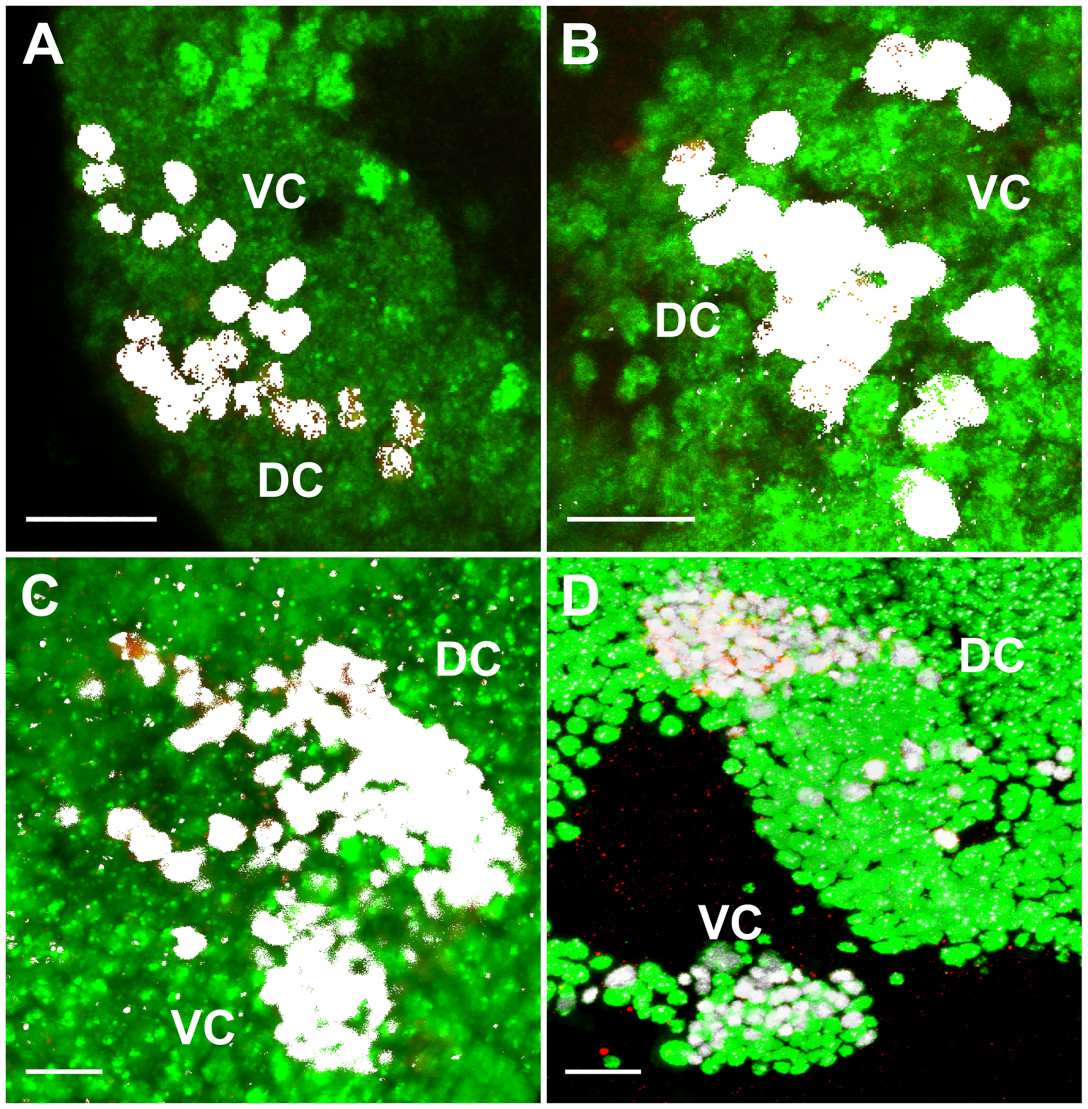
Cells expressing Poxn in larval and adult brains are post-mitotic neurons. (**A–D**) Colocalization (white) in nuclei of Poxn (red) and Elav (green) proteins, visualized by immunofluorescent staining, is shown in wild-type (*Ore-R*) brain hemispheres of first (A), second (B), and late third instar larvae (C), and of adults (D). Staining of all *Poxn*-nuclei with Elav was corroborated by visual inspection in single layers of the Z-stacks. Panels show maximum intensity projections of CLSM sections of Z-stacks extending over 43 μm (A), 45 μm (B), and 50 μm (C) at 63x magnification, and of a Z-stack extending over 26 μm at 40x magnification (D). Scale bars: 10 μm (A–C) and 20 μm (D).

Throughout development, all cells of the developing brain that express Poxn also express Elav (Fig 2, S2A and S3 Figs), an early marker of neuronal differentiation required throughout neuronal development [43], which suggests that they are all post-mitotic neurons. Consistent with this observation, none of them expresses Repo (S4 Fig), a marker for differentiating glia [44,45]. Some of these neurons that express Poxn—henceforth referred to as *Poxn*-neurons, even if, like in *Poxn* mutants, no Poxn protein is expressed—begin to extend neurites by stage 14 of embryogenesis (S3 Fig). According to the time when they begin to express Poxn in wild-type brains, we divide *Poxn*-neurons into two classes, embryonic and (third instar) larval *Poxn*-neurons. In late third instar brains, Prospero (Pros), which acts as binary switch between neural stem cells and terminally differentiating neurons [46,47,48], is expressed in most *Poxn*-neurons (S5A–S5C Fig). Careful analysis of individual layers of the Z-stack shows that only 20 *Poxn*-neurons of the DC and 12 of the VC do not express Pros (S5D–S5F, S6, S7A and S7B Figs). These are the 32 embryonic *Poxn*-neurons since, in contrast to postembryonic neurons [49], Pros is not expressed in neurons of the embryonic brain [50,51]. All *Poxn*-neurons, except the 32 embryonic *Poxn*-neurons, still have no neurites (S5D–S5F Fig). As shown below, larval *Poxn*-neurons begin to differentiate neurites only during metamorphosis.

### Embryonic *Poxn*-neurons have larger nuclei than larval *Poxn*-neurons

Strikingly, the 32 embryonic *Poxn*-neurons, which show no expression of Pros, have the largest nuclei among all *Poxn*-neurons present in late third instar brains, both in the DC and VC, and thus can be identified not only on the basis of the absence of Pros but also roughly by their large nuclear diameters because larval and embryonic *Poxn*-neurons show very little overlap with regard to nuclear size (S7A and S7B Fig). These differences in nuclear size are not small, as nuclear diameters vary by a factor of 1.6 (VC) to 1.7 (DC), corresponding to a four- to fivefold difference in nuclear volume (S7A and S7B Fig). In addition, there is a striking bias in nuclear size of *Poxn*-neurons along the anteroposterior axis, with large nuclei closer to the posterior in both the DC and VC (S7C and S7D Fig).

Interestingly, this difference in nuclear size may still be evident in the adult brain since there is a clear bimodal nuclear size distribution for neurons in the adult brain that begin to express *Poxn* during embryogenesis or only during third instar (S8A and S8B Fig). Indeed, during metamorphosis the average nuclear diameter of the embryonic *Poxn*-neurons increases from 3.4 ± 0.15 μm to 4.3 ± 0.23 μm (s.d.; computed from the data plotted in S7A, S7B and S8A, S8B Figs), which corresponds to a twofold increase in volume. Moreover, the bias in nuclear diameters along the anteroposterior axis of late third instar larval brains is still observed in the DC and VC of adult brains, though less pronounced (S8C and S8D Fig). A similar change in cell-body diameter, when moving from more deeply located early born embryonic neurons to later born neurons in more superficial layers, has been ascribed to a transition in expression from Chinmo to Br-C [52]. This is consistent with our observations, as the plot from anterior to posterior in S8 Fig parallels one from superficial to deeper layers.

### Embryonic and larval *Poxn*-neurons constitute virtually all *Poxn*-neurons of the adult brain

**Fig 3:**
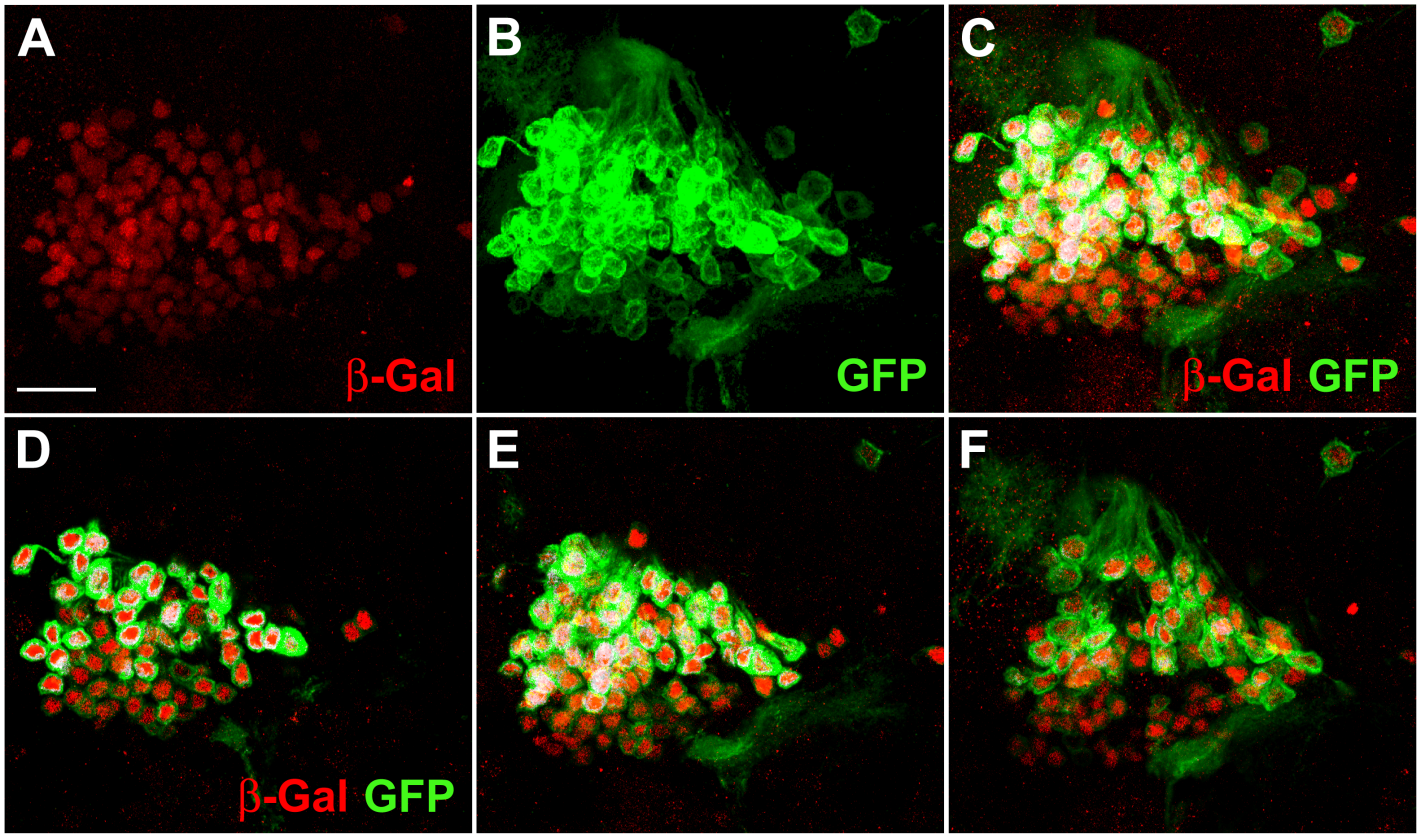
Most *Poxn*-neurons survive metamorphosis and continue to express Poxn in the adult brain. (**A–C**) The DC of *Poxn*-neurons, visualized by immunofluorescent staining for β-Gal (red) and GFP (green), is shown in the brain of a *w*^*1118*^ *UAS-Flp/*+; *tub-Gal80*^*ts*^*/+*; *Act5C>polyA>lacZ*.*nls1 Poxn-Gal4-13-1/Poxn-CD8*::*GFP* female. A maximum intensity projection of the same CLSM sections of an entire Z-stack is shown in the red channel (A), green channel (B), and in both channels (C) at 63x magnification. β-Gal-positive nuclei (red) belong to cells that expressed Poxn at the time of heat shock during the third larval instar (feeding stage). The membrane-associated CD8::GFP fusion protein labels Poxn-expressing cells at the time of fixation. (**D**–**F**) Maximum intensity projections of substacks at 0–5 μm (D), 5–10 μm (E), and 10–18 μm (F) of the Z-stack extending from 0 (anterior) to 31 μm (posterior) shown in (C), which includes all *Poxn*-nuclei. Virtually all β- Gal-labeled neurons also express GFP. Similar results were obtained for the VC of *Poxn*-neurons (data not shown). Scale bar: 20 μm.

To determine the number of *Poxn*-neurons at the end of the third instar is difficult and cannot answer the question whether these correspond to those of the adult brain since it is conceivable that some *Poxn*-neurons do not survive to adulthood or have ceased to express Poxn, while new *Poxn*-neurons appear during metamorphosis. To examine this question, *Poxn*-neurons were specifically labeled by Flippase that permanently activated *lacZ* encoding nuclear β-Gal. Specific activation of Flippase in *Poxn*-neurons was achieved through the activation of *UAS-Flp* by *Poxn-Gal4* at an elevated temperature that reduces the inhibition of Gal4 by the ubiquitously expressed Gal80^ts^ (see Materials and methods). Accordingly, *UAS-Flp*/(+ or *Y*); *tub-Gal80*^*ts*^/+; *Act5C>polyA>lacZ*.*nls1 Poxn-Gal4*/*Poxn-CD8*::*GFP* third instar feeding larvae were heat shocked for 6 hours at 32°C. The *Poxn-CD8*::*GFP* transgene, which expresses the CD8::GFP fusion protein under control of the *Poxn* upstream region that includes the *Poxn* brain enhancer, labels *Poxn*-neurons in the brain throughout development. No *Poxn*-neurons can be labeled by β-Gal before they begin to express *Poxn*, and *UAS-Flp* is again repressed by Gal80^ts^ at the permissive temperature of 18°C after the heat shock. Therefore, *Poxn*-neurons that would appear only during metamorphosis would not be labeled by β-Gal. No such *Poxn*-neurons appear to be visible in the adult brain (Fig 3). On closer inspection of single layers of entire confocal stacks, only one such *Poxn*-neuron each was observed in two DCs of adult brains, while three cells that express β-Gal in each of the two DCs examined seem not to be labeled by GFP, which indicates that perhaps a few cells cease to express Poxn during metamorphosis. Similarly, in VCs only one *Poxn*-neuron appeared not to be labeled by β-Gal, and only one cell expressed β-Gal in each of two VCs but was not labeled by GFP.

Thus, virtually all cells of the DC and VC express both β-Gal and CD8::GFP, which suggests that all *Poxn*-neurons in the brain of late third instar larvae survive metamorphosis and continue to express Poxn. Conversely, all *Poxn*-neurons of the adult brain have initiated Poxn expression by late third instar.

### Projection patterns of *Poxn*-neurons during larval development

**Fig 4:**
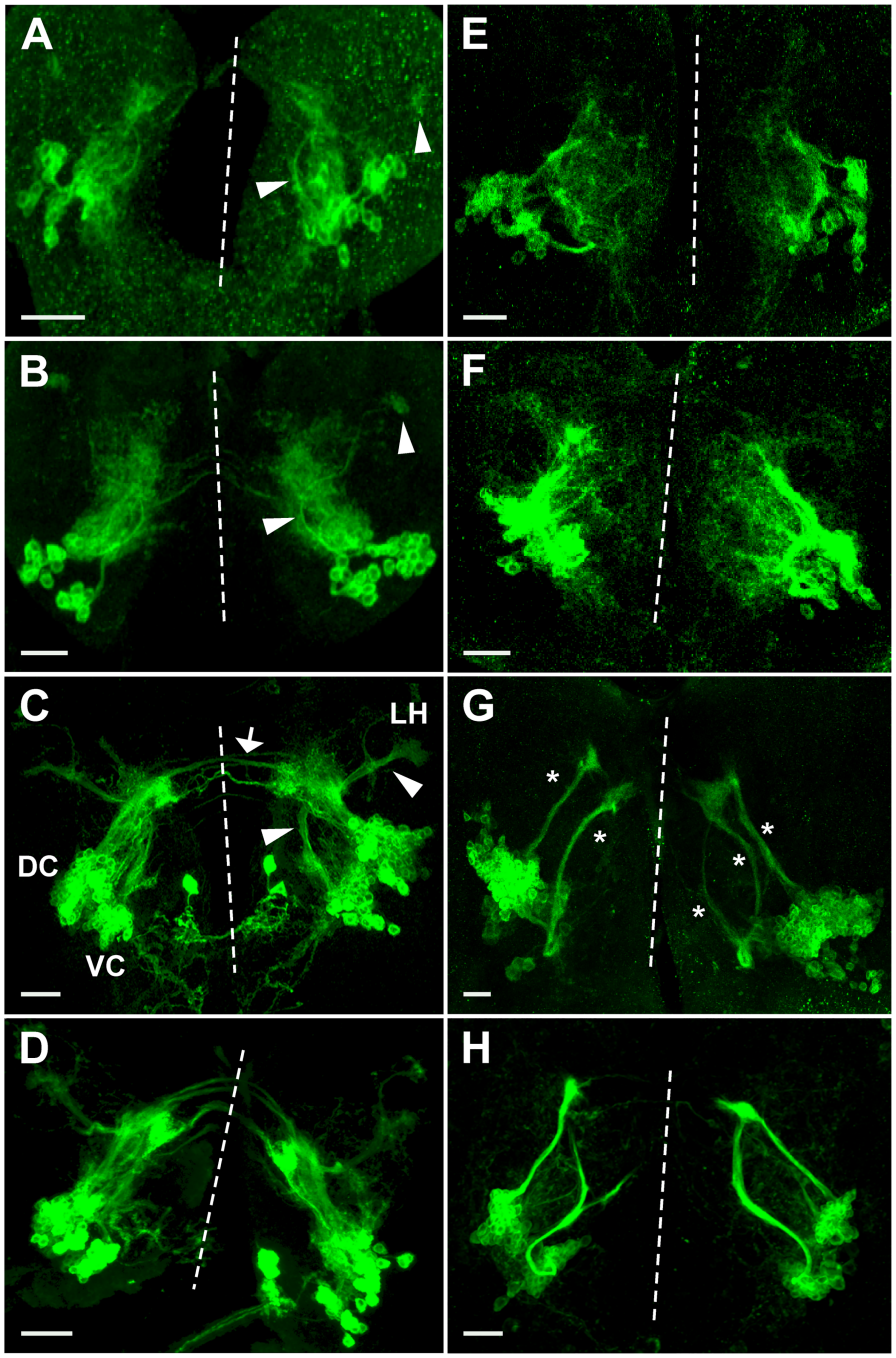
Projection patterns of *Poxn*-neurons in wild type and *Poxn* mutants during larval development. (**A–H**) *Poxn*-neurons are visualized by the expression of *Poxn-CD8*::*GFP* and immunofluorescent staining for GFP in first (A,E), late second (B,F), and late third instar (C,D,G,H) brains of *w*^*1118*^; *Poxn-CD8*::*GFP* (A-C), *w*^*1118*^; *Poxn*^*ΔM22-B5*^; *Poxn-CD8*::*GFP* (E-G), *w*^*1118*^; *Poxn*^*ΔM22-B5*^ *Poxn-SuperA-158*; *Poxn-CD8*::*GFP* (D), and *w*^*1118*^; *Poxn*^*ΔM22-B5*^ *Poxn-Sbl-107*; *Poxn-CD8*::*GFP* (H) larvae. Note that the projection patterns of the latter two resemble those in wild-type (C) and *Poxn* mutant brains (G), respectively. Arrowheads in (A-C) point to arc-like projections of the VC and their targets in the lateral protocerebrum, and arrow in (C) points to tracts of the SEC, emanating from the DC of wild-type brains. Asterisks in (G) mark aberrant projections from both *Poxn* clusters in a *Poxn* mutant brain. Dashed lines indicate midlines of the flattened brains viewed along the anteroposterior axis. Panels show maximum intensity projections of CLSM sections of Z-stacks extending over 41 μm (A), 50 μm (B), 53 μm (C), 49 μm (D), 75 μm (E), 36 μm (F), 48 μm (G), and 77 μm (H) at 20x magnification. Panel H shows the same brain as Fig 6B. DC, dorsal cluster of *Poxn*-neurons; LH, lateral horn; VC, ventral cluster of *Poxn*-neurons. Scale bars: 20 μm.

Some *Poxn*-neurons of the DC differentiate neurites and extend axons that meet at the midline of the SEC already by stage 14 of embryogenesis, while *Poxn*-neurons of the VC show no overt differentiation of neurites at this stage (S3 Fig). However, in first instar larval brains also *Poxn*-neurons of the VC extend neurites that project in an arc toward the anterior lateral brain regions (Fig 4A). These distinct projection patterns of the DC and VC are also observed in second instar larval brains (Fig 4B), which suggests that the embryonic *Poxn*-neurons are grouped into two populations, DC and VC (S2 Fig), which are specified for different functions already by these early larval stages. During third instar, the projections of the embryonic *Poxn*-neurons follow the paths established during embryogenesis and the first two instars, while the density of their neurites increases considerably (Fig 4C). The neurites of the DC project towards the dorsomedial part of the brain, and some extend tracts into the SEC. The projections from the VC follow an arc-like path and target the LH (Fig 4C). Only the 32 embryonic *Poxn*-neurons, which are free of Pros, form neurites during embryogenesis and larval stages (S5 Fig), whereas the larval *Poxn*-neurons are not yet engaged in these processes.

**Fig 5:**
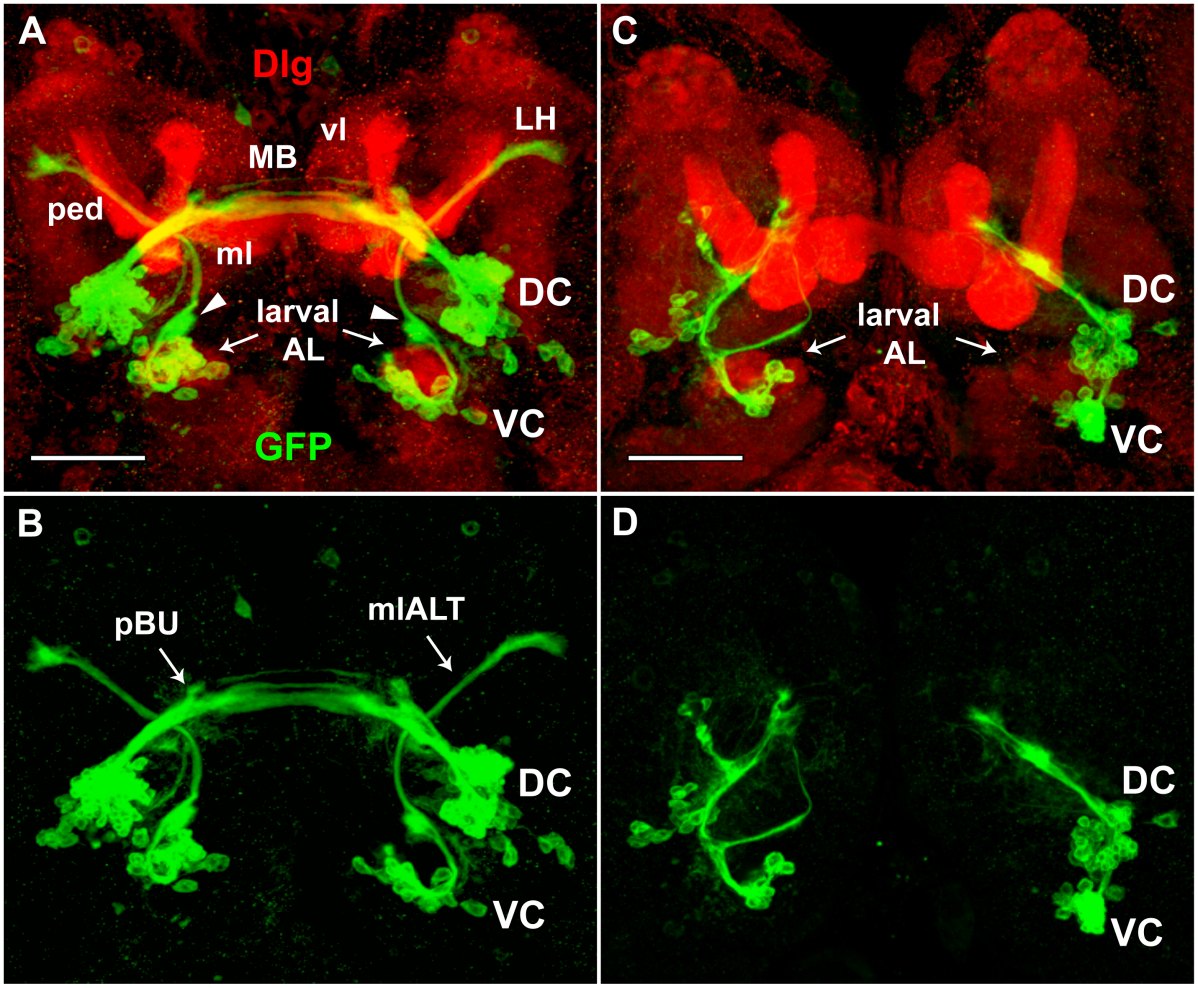
Projections of *Poxn*-neurons do not express Dlg in wild-type and *Poxn* mutant brains of late third instar larvae. *Poxn*-neurons, immunostained for the expression of *Poxn-CD8*::*GFP* (green), and neuropils, immunostained for the expression of Dlg (red), are shown in brains of *w*^*1118*^; *Poxn-CD8*::*GFP* **(A,B)** and *w*^*1118*^; *Poxn*^*ΔM22-B5*^ *Poxn-Sbl-107*; *Poxn-CD8*::*GFP* **(C,D)** late third instar larvae, in the red and green channel (A,C) and only in the green channel (B,D) at 40x magnification. To improve the visibility of the axon tracts and neuropils, especially the larval ALs, substacks extending from 26 μm (anterior) to 72 μm (posterior) (A,B) and from 18 μm (anterior) to 67 μm (posterior) (C,D) of Z-stacks, extending over 87 μm, are shown as maximum intensity projections of CLSM sections. Although these substacks exclude many cell bodies of the *Poxn*-neurons visible in the excluded substacks, they include all neurite projections. The mlALT originating from the VC crosses behind the commissural tracts emanating from the DC. Arrowheads in (A) point at ‘swellings’ of the mlALT preceding dendrite formation in the region of the future AL. Screening through single stacks shows no colocalization of GFP and Dlg in (A) and (C). DC and VC, dorsal and ventral cluster of *Poxn*-neurons; larval AL, larval antennal lobe; LH, lateral horn; mlALT, mediolateral antennal lobe tract; MB, larval mushroom bodies; vl, vertical lobe of MB; ml, medial lobe of MB; ped, pedunculus of MB; pBU, primordial bulb. Scale bars: 50 μm.

**Fig 6:**
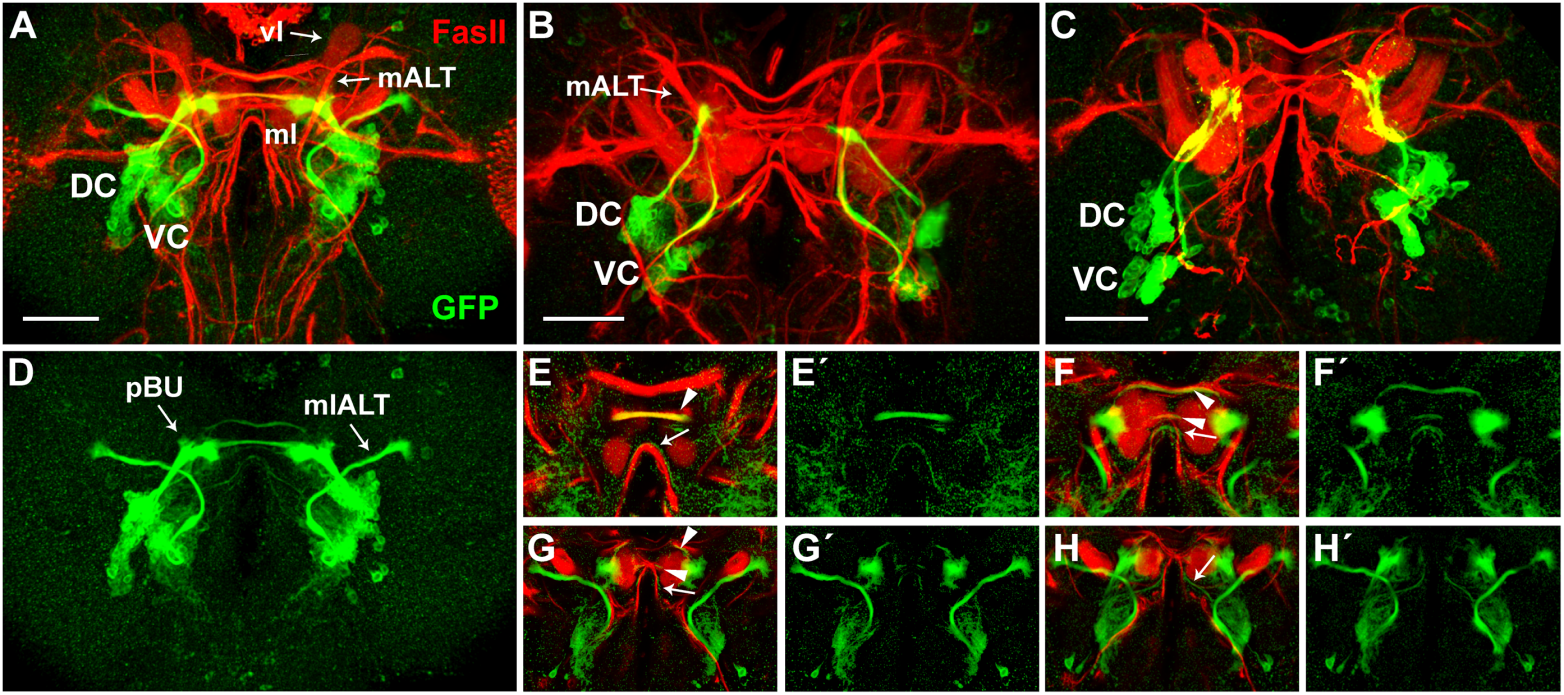
Projections of *Poxn*-neurons and axonal tracts labeled by FasII in wild-type and *Poxn* mutant brains of late third instar larvae. **(A-D)** *Poxn*-neurons, immunostained for the expression of *Poxn-CD8*::*GFP* (green), and neuropils, immunostained for the expression of FasII (red), are shown in brains of *w*^*1118*^; *Poxn-CD8*::*GFP* **(A,D)** and *w*^*1118*^; *Poxn*^*ΔM22-B5*^ *Poxn-Sbl-107*; *Poxn-CD8*::*GFP* **(B,C)** late third instar larvae in the red and green channel (A-C) and only in the green channel (D) at 40x magnification. The two *Poxn* mutant brains display the two types of mutant projection patterns commonly observed: projections from the VC seem to follow the mALT instead of the mlALT (B), or adopt an entirely different path, eventually running parallel to the projections from the DC before they stall (C). Scale bars: 50 μm. **(E-H')** Central parts of substacks of (A) are shown from posterior to anterior at 10–15 μm (E,E'), 23–28 μm (F,F'), 28–30 μm (G,G'), and 30–35 μm (H,H') as maximum intensity projections of CLSM sections of a Z-stack extending over 85 μm in both channels (E-H) and only in the green channel (E'-H') at 40x magnification. *Poxn*-neuron tracts that pass through the SEC and do or do not co-express FasII are indicated by arrowheads and arrows, respectively. DC, dorsal cluster of *Poxn*-neurons; vl, vertical lobe of mushroom bodies; mALT, middle antennal lobe tract; mlALT, mediolateral antennal lobe tract; ml, medial lobe of mushroom bodies; pBU, primordial bulb; VC, ventral cluster of *Poxn*-neurons.

To map the neurite projection patterns of the embryonic *Poxn*-neurons with regard to neuropils in late third instar larval brains, the postsynaptic marker Discs-large (Dlg) [53,54,55] and the axon tract marker Fasciclin II (FasII) [56,57] were used. Maximum intensity projections of *Poxn*-neurons colabeled with Dlg (Fig 5A) or FasII (Fig 6A) do not resolve position along the anteroposterior axis and hence cannot decide whether the labeled proteins are expressed in the same cells. Careful analysis of individual layers of confocal Z-stacks, however, reveals that there is no colabeling of *Poxn*-neurons with Dlg. In particular, the postsynaptic Dlg is not expressed in *Poxn*-neurons at the larval ALs or the primordial bulbs, where it would be expected if synapses had formed at these larval neuropils. The absence of synapses at larval neuropils was corroborated similarly by immunolabeling the presynaptic markers Synapsin, regulating the release of neurotransmitters, and nc82 [58], the Bruchpilot protein present at active synapses [59]. Neither of these proteins was expressed in *Poxn*-neurons (our unpublished results), in particular not at the future LHs or in the SEC where they will form their presynaptic endings in the adult neuropils of the LHs and EB during metamorphosis.

Analysis of individual layers of confocal stacks further shows that projections, emanating from the DC as two, or perhaps three, thick closely apposed axon bundles, split into several tracts at the primordial bulb (pBU), and run in a dorsal and posterior direction along the SEC (Fig 5A and 5B). The three ventral, thicker tracts (Fig 5B) run smoothly along, and immediately dorsal and posterior to, the medial lobes of the larval MB (Fig 5A), while the most dorsal tract at the midline of the commissure is slightly anterior to the other tracts. This tract appears to originate from a separate more ventral, thinner axon bundle of the DC, which passes in front of the thick bundles at the pBU (Fig 6D). Projections of the VC run dorsomedially around the larval AL and then bend dorsolaterally in an arc-like path, the mlALT, to target the LH (Fig 5A and 5B). As characterized previously [15,16,17], the mlALT is specific for ventral PNs (vPNs). In the region where the mlALT leaves the larval AL, it bifurcates into a dorsal and ventral tract (Fig 5A and 5B). While the dorsal tract continues along the mlALT, the ventral tract stops and forms a ‘swelling’ where the adult AL will develop during metamorphosis (arrowheads in Fig 5A). A slight ‘swelling’ of the mlALT at this position is evident as well. These ‘swellings’ of the dorsal and ventral tracts may constitute short dendrites that precede arborization of adult vPNs and LNs, respectively, at the AL during metamorphosis (see below). The mlALT remains in a relatively narrow layer perpendicular to the anteroposterior axis of the brain (Fig 6G' and 6H'), and, after crossing the axon bundles emanating from the DC immediately posterior to them, partly runs smoothly along and posterior to the pedunculus of the MB, which expresses Dlg (Fig 5A).

Whereas none of these axon bundles of embryonic *Poxn*-neurons label with Dlg, some are labeled by FasII (Fig 6A), as revealed by analysis of single confocal layers. The bundled projections extending from the DC do not co-label with FasII up to the pBU, where they express FasII. At the pBU they split into several tracts, of which the three most dorsal tracts label with FasII (Fig 6E–6G), whereas the two ventral thin tracts do not (Fig 6E–6H). In contrast, the mlALT emanating from the VC is not labeled by FasII (Fig 6G and 6H). Throughout larval development, the projections of the *Poxn*-neurons of the VC follow the mlALT, which appears as a tight bundle between the cell bodies and the terminals (Figs 4A–4C, 5A and 5B). During third instar, the thickness of the projections passing through the mlALT and the neurites of the *Poxn*-neurons at the LH increase in number and volume (compare Figs 4C, 5B and 6D with Fig 4B).

**Fig 7:**
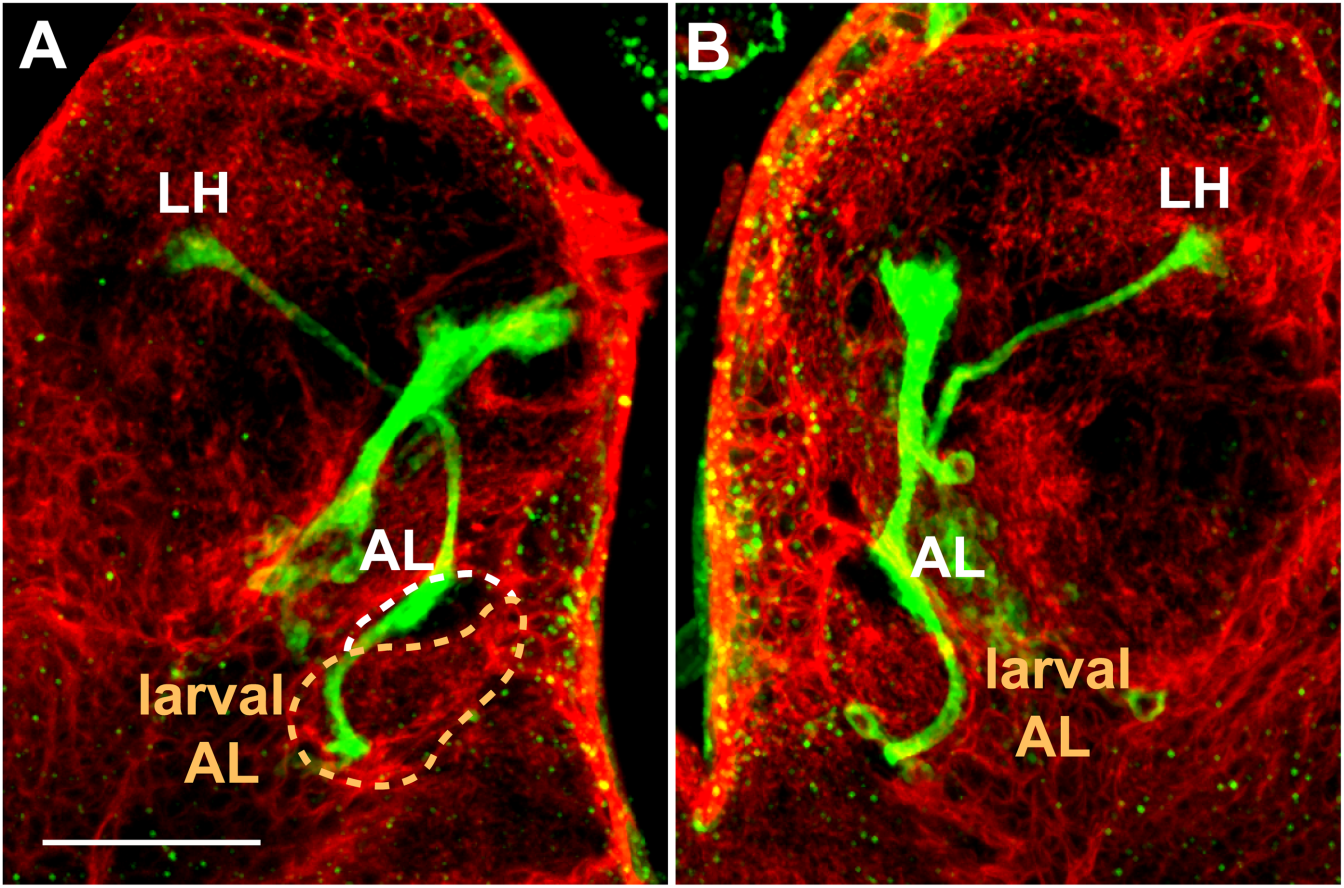
Projection pattern of *Poxn*-neurons with respect to glia in late third instar larval brain. (**A,B**) Left (A) and right (B) hemispheres of *w*^*1118*^; *UAS-CD2*; *repo-Gal4/Poxn-CD8*::*GFP* late third instar larval brain, immunostained for GFP (green), labeling *Poxn*-neurons, and CD2 (red) in glial membranes [60]. The axons of the *Poxn*-neurons project along the lateral region of the larval AL (surrounded by dashed orange line in A), exhibit a striking ‘swelling’ at the developing adult AL (surrounded by dashed white line) but no arborization, and follow the mlALT to target the LH. No arborization is detected at the larval AL. To optimize the visibility of the axon tracts of the *Poxn*-neurons from the VC along the larval AL and developing adult AL to the LH, substacks extending from 30 μm (anterior) to 56 μm (posterior) (A) and from 48 μm (anterior) to 76 μm (posterior) (B) of a Z-stack, extending over 85 μm, are shown as maximum intensity projections of CLSM sections at 40x magnification. Although these substacks exclude the cell bodies of the *Poxn*-neurons, they include all neurite projections. Scale bar: 50 μm.

The AL begins to develop adjacent and dorsal to the larval AL and by late third instar is recognized as a ‘swelling’ of the mlALT (Figs 5A, 5B, 6A, 6D and 7). Although the AL is not yet labeled by the presynaptic neuropil marker mAb nc82 [58], it can be visualized by the membranes of the surrounding glia, marked with *repo-Gal4* driving *UAS-CD2* expression (Fig 7). The processes of *Poxn*-neurons of the VC pass along the lateral region of the larval AL, the developing AL, and the mlALT to target the LH (Fig 7). The projections show no arborization at the larval AL.

### Projection patterns of *Poxn*-neurons in *Poxn* mutant larvae

The number of *Poxn*-neurons in mutant and wild-type larval brains of the same instar are similar although a precise determination of their number in third instar *Poxn*^*ΔM22-B5*^; *Poxn-CD8*::*GFP* mutant brains is impossible (Table 1). However, their projection patterns in *Poxn* mutant brains are drastically altered at all larval stages (Fig 4E–4H). The bilateral symmetry of their projections is disturbed throughout larval development (compare Fig 4E–4G with Fig 4A–4C). This is true also for *Poxn*-neurons in mutants whose *Poxn* functions except the brain function have been rescued by a *Poxn-Sbl* transgene (Fig 4H) as compared to *Poxn*-neurons in mutants rescued by a complete *Poxn* transgene (Fig 4D). The typical arc-like projections emanating from the VC in the wild type (arrowheads in Fig 4A–4C) are missing in mutants (Fig 4E–4G). The neurites lack a specific orientation and are diffusely directed towards the midline in first and second instar larvae (Fig 4E and 4F). During third instar, the *Poxn*-neurons form two discernable clusters, and their neurites fasciculate, generating two, sometimes three, bundles that emanate in the dorsomedial direction (Fig 4G). Most strikingly, the axon bundles stall in the regions, where in the wild type they finally turn towards their targets (Fig 4C), and form neurites at their distal ends (Fig 4G and 4H). Accordingly, only very few projections appear in the SEC and none at the LH (Fig 4G and 4H).

Similar results were obtained after labeling synapses with Dlg (Fig 5C and 5D) or long axons with FasII (Fig 6B and 6C). Projections from the VC bypass the larval AL (Fig 5C), begin to follow the medial antennal lobe tract (mALT) [27], formerly called inner antennocerebral tract (iACT) [15] (Fig 6B), or run parallel to the projections emanating from the DC and turn dorsally behind the larval AL (Fig 6C), but then are stalled and thus fail to target the LH (Fig 5C). Similarly, projections from the DC stall and fail to project into the SEC (Figs 5C, 5D and 6B, 6C).

### Formation of ellipsoid body and bulb in the pupal brain

**Fig 8:**
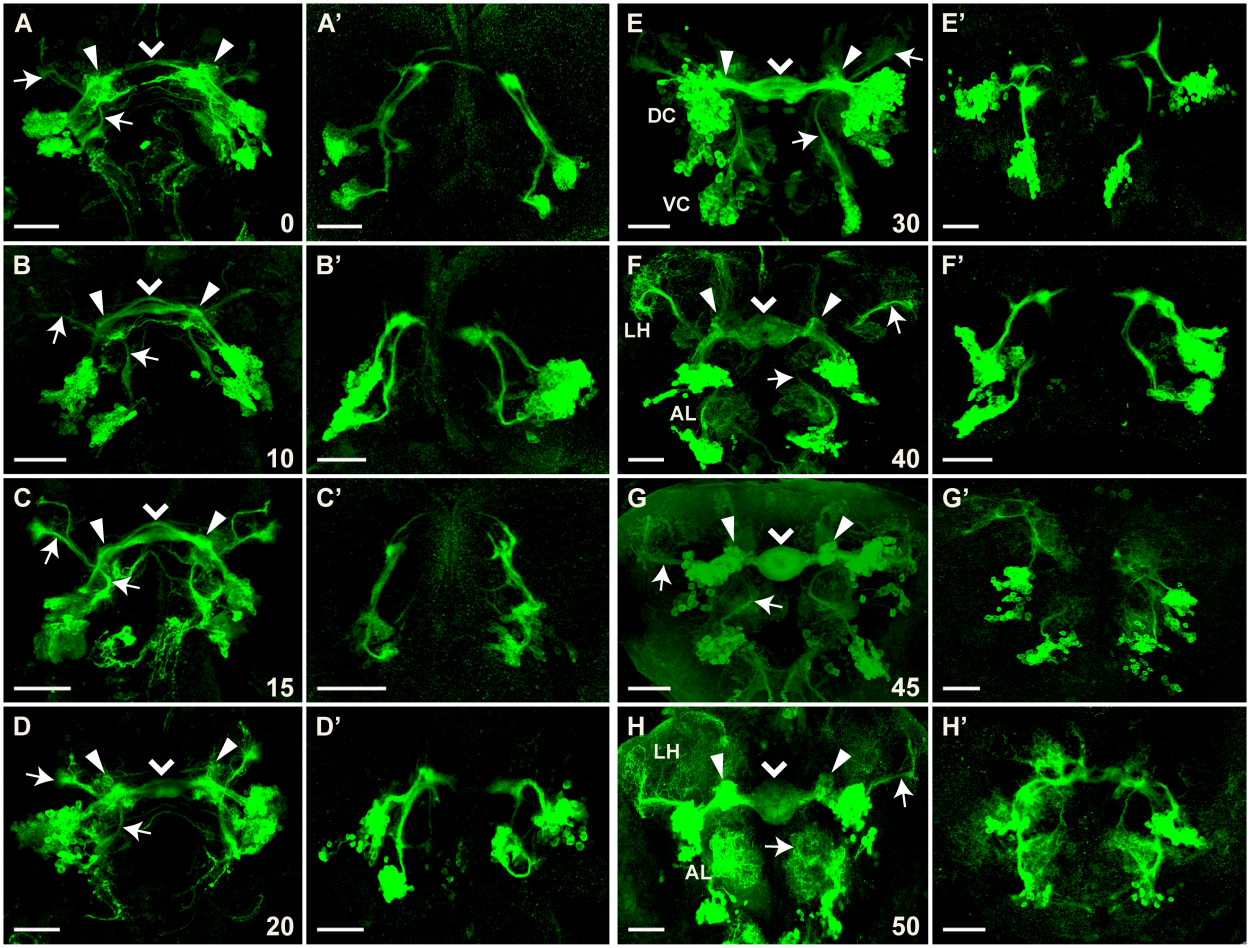
Formation of ellipsoid body depends strongly on *Poxn* function. Brains of *w*^*1118*^; *Poxn-CD8*::*GFP* (**A-H**) or *w*^*1118*^; *Poxn*^*ΔM22-B5*^ *Poxn-Sbl-107*; *Poxn-CD8*::*GFP* (**A’-H’**) pupae, immunostained for GFP at 0 h APF (A,A’), 10 h APF (B,B’), 15 h APF (C,C’), 20 h APF (D,D’), 30 h APF (E, E’), 40 h APF (F,F’), 45 h APF (G,G’), and 50 h APF (H,H’), are shown as maximum intensity projections of confocal Z-stacks at 20x magnification (panels except E, G, and G’ show substacks that remove some of the cell bodies but improve the visibility of projections). Z-stacks extended over 89 μm (A), 67 μm (B), 82 μm (C), 52 μm (D), 72 μm (E), 54 μm (F), 79 μm (G), 51 μm (H), 44 μm (A’), 65 μm (B’), 90 μm (C’), 88 μm (D’), 56 μm (E’), 46 μm (F’), 102 μm (G’), and 66 μm (H’). Arrows point to the bilateral arc-like mlALTs visible at all pupal stages. Filled arrowheads point to the arborizations at the developing bulbs, while arrowheads point to the midline of the forming ellipsoid body neuropil. Note that arborizations at the lateral horn (LH) and antennal lobe (AL) become prominent around 40 h APF in the wild-type brain (F-H), while they are slightly delayed in mutant brains where they occur at the end of the stalled projections (G’,H’). Pupal stages, measured as hours APF, imply a developmental temporal variation corresponding to about 5%. Scale bars: 50 μm.

At late third instar (Fig 4C) and the onset of pupariation (Fig 8A), the embryonic *Poxn*-neurons of the DC extend their neurites into the SEC. Thereafter, larval *Poxn*-neurons begin to form neurites and the commissural neurites increase greatly in number, follow the specific paths of persistent larval projections of the embryonic *Poxn*-neurons (Fig 8B and 8C), and by 20–30 h APF give rise to a kidney-like shape of the forming EB (Fig 8D and 8E). The two clusters of *Poxn*-neurons separate and are easily distinguished as DC and VC by 30 h APF (Fig 8E). The kidney-shaped structure of the EB is now obvious and retains this shape till about 40 h APF (Fig 8F). By 45–50 h APF, it is transformed into the doughnut-like shape characteristic of the adult EB (Fig 8G and 8H). The DC neurons reveal compact arborizations in the bulbs (Fig 8H; [10,14,61]) that seem to originate during the third larval instar (Figs 5B and 6D) and continue to grow during pupal stages (Fig 8A–8H). The excessive gain in volume and density of arborizations from DC neurons during pupal development cannot be attributed to an increase in *Poxn*-neurons, as their number does not change during metamorphosis (Table 1).

### Developing ventral *Poxn*-neurons in the pupal brain

Similar to the *Poxn*-neurons of the DC, the *Poxn*-neurons of the VC send their processes along the paths established by the embryonic *Poxn*-neurons during larval stages through the mlALT (Fig 4A–4C), which maintains its arc-like shape, and continue to target the developing AL and LH throughout pupal stages (Fig 8A–8H). In contrast to the third instar, when the VC displays arborizations at the LH but none in the AL region (Figs 4C and 7), dendritic arbors begin to form at the developing AL and increase in density during pupal stages (Fig 8A–8E), becoming profuse by 40–50 h APF (Fig 8F–8H).

### Developing *Poxn*-neurons in the *Poxn* mutant pupal brain

*Poxn*-neurons of wild-type and *Poxn* mutant pupal brains are comparable in number and positions. Until 40 h APF, the *Poxn*-neurons of the DC display phenotypes (Fig 8A’–8F’) similar to those observed in late third instar larvae (Figs 4G, 4H, 5C, 5D, 6B and 6C). Most of the neuronal processes of the DC neurons remain stalled and fail to turn towards the midline. Thus, the projections through different commissural tracts, evident in the wild type already during early and late larval stages (Fig 4A–4C) or in early pupae (Fig 8A–8C), and the kidney-shaped neuropil, formed between 20 and 40 h APF in wild-type pupal brains (Fig 8D–8F), fail to develop in mutant pupal brains (Fig 8A’–8F’). After 45 h APF, several neuronal processes appear to extend into the SEC but display an irregular morphology that does not resemble the EB (Fig 8H’). Similar to wild-type brains, however, relatively little arborization appears before 40 h APF. Profuse arborization is slightly delayed in mutant brains and occurs aberrantly at the stalled projections (Fig 8G’ and 8H’).

The neurites of the VC neurons project in aberrant directions, a behavior similar to that of DC neurons at all pupal stages (Fig 8A’–8H’). Until 40 h APF, these processes form thick bundles that stall in the same region as those of the DC neurons (Fig 8A’–8F’). After 40 h APF, these projections show diffuse arborizations (Fig 8G’ and 8H’). Arborization at the developing adult AL is far less prominent in mutant (Fig 8G’ and 8H’) than in wild-type brains (Fig 8F–8H). Like the LH of mutant larvae, the developing LH is not targeted in mutant pupae, and thus the *Poxn*-neurons of the VC do not contribute to the mlALT at any stage of pupal brain development in *Poxn* mutants (Fig 8A’–8H’).

### *Poxn*-neurons in the wild-type adult brain

**Fig 9:**
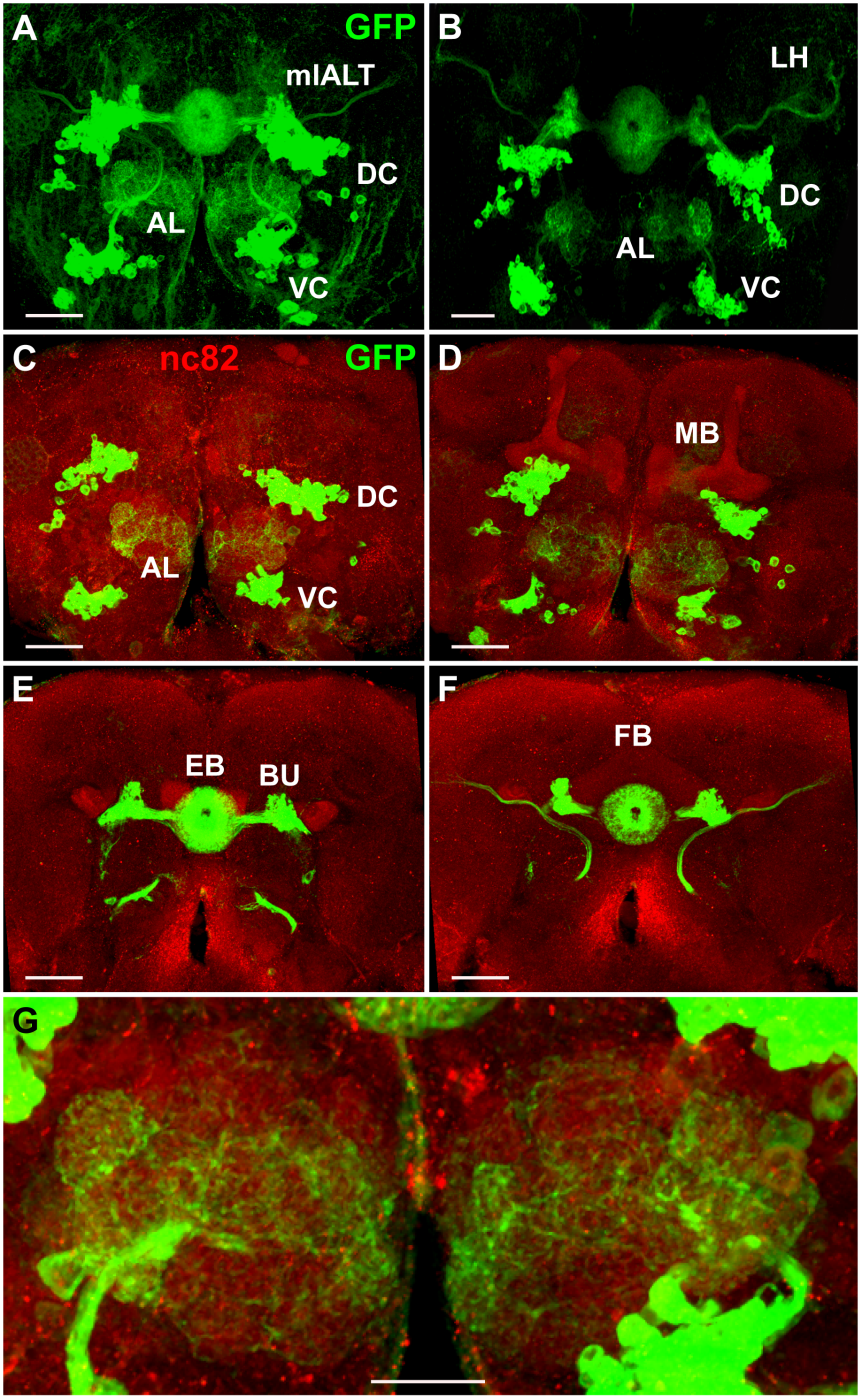
Projection pattern of *Poxn*-neurons in adult brains. **(A,B**) Projections of Poxn-neurons in *w*^*1118*^; *Poxn-CD8*::*GFP* (A) and *w*^*1118*^; *Poxn*^Δ*M22-B5*^ *Poxn-SuperA*; *Poxn-CD8*::*GFP* (B) adult brains (dorsal side up), immunostained for GFP, are shown as maximum intensity projections of Z-stacks, extending over 76 μm at 40x magnification (A) and extending over 75 μm at 20x magnification (B). Projection patterns in the brain of *Poxn* mutants rescued by the complete *Poxn* transgene *Poxn-SuperA* (B) are similar to those in the wild-type brain (A). (**C**–**F**) Substacks of (A), immunostained for GFP (green) and nc82 (red), are shown from anterior to posterior at 6–14 μm (C), 14–25 μm (D), 34–43 μm (E), and 43–66 μm (F). Note that what may look like cell bodies at the BU in (A,B,E,F) are the bush-like postsynaptic arborizations of the *Poxn*-R neurons at the glomeruli of the ipsilateral BU [10,14,61]. (**G**) Enlarged region of substacks of (A) at 6–35 μm, illustrating the dendritic arborizations of *Poxn*-neurons invading many glomeruli of the ALs. AL, antennal lobe; DC, dorsal cluster; EB, ellipsoid body; FB, fan-shaped body; LH, lateral horn; BU, bulb; MB, mushroom bodies; mlALT, mediolateral antennal lobe tract; VC, ventral cluster. Scale bars: 50 μm (A–F) and 25 μm (G).

Most, if not all, adult *Poxn*-neurons of the DC are large-field R neurons, forming concentric ring-shaped arbors in the EB [10,14,61]. This is evident from their morphology and colocalization of Poxn protein with the expression of enhancer trap lines specific for various subpopulations (R1–R4) of R neurons ([14]; Fabian Schmid, WB, MN, unpublished results). The ring-shaped arbors of the *Poxn*-neurons are more pronounced in the adult (Fig 9A, 9B, 9E and 9F) than in the pupal brain at 50 h APF (Fig 8H). In addition to the EB, the DC *Poxn*-neurons target only the bulb (Fig 9A, 9B, 9E and 9F) where they form bush-like dendritic arbors [10,14,61]. The *Poxn*-neurons do not display any obvious sexual dimorphism (data not shown). The projection patterns of *Poxn*-neurons in brains of *Poxn* mutants rescued by a *Poxn* transgene (Fig 9B) that carries all *Poxn* functions, *Poxn-SuperA* ([19]; see S1 Fig), are very similar to those in wild-type brains (Fig 9A), an observation consistent with the analysis of adult brains by paraffin sections. In these sections, the EB is clearly visible and similar in wild-type and mutant brains rescued by *Poxn*-*SuperA* (compare S9A and S9C Fig with S9B and S9D Fig).

Like in third instar larvae and in pupae, VC neurons project in the adult through the major long axon tract, the mlALT (Fig 9A and 9B). Their dendritic projections invade many glomeruli of the AL, recognizable by staining for the synaptic marker nc82 (Fig 9C, 9D and 9G), while their axonal projections pass through the mlALT to target the LH (Fig 9A, 9B and 9F).

### *Poxn*-neurons in the *Poxn* mutant adult brain

**Fig 10:**
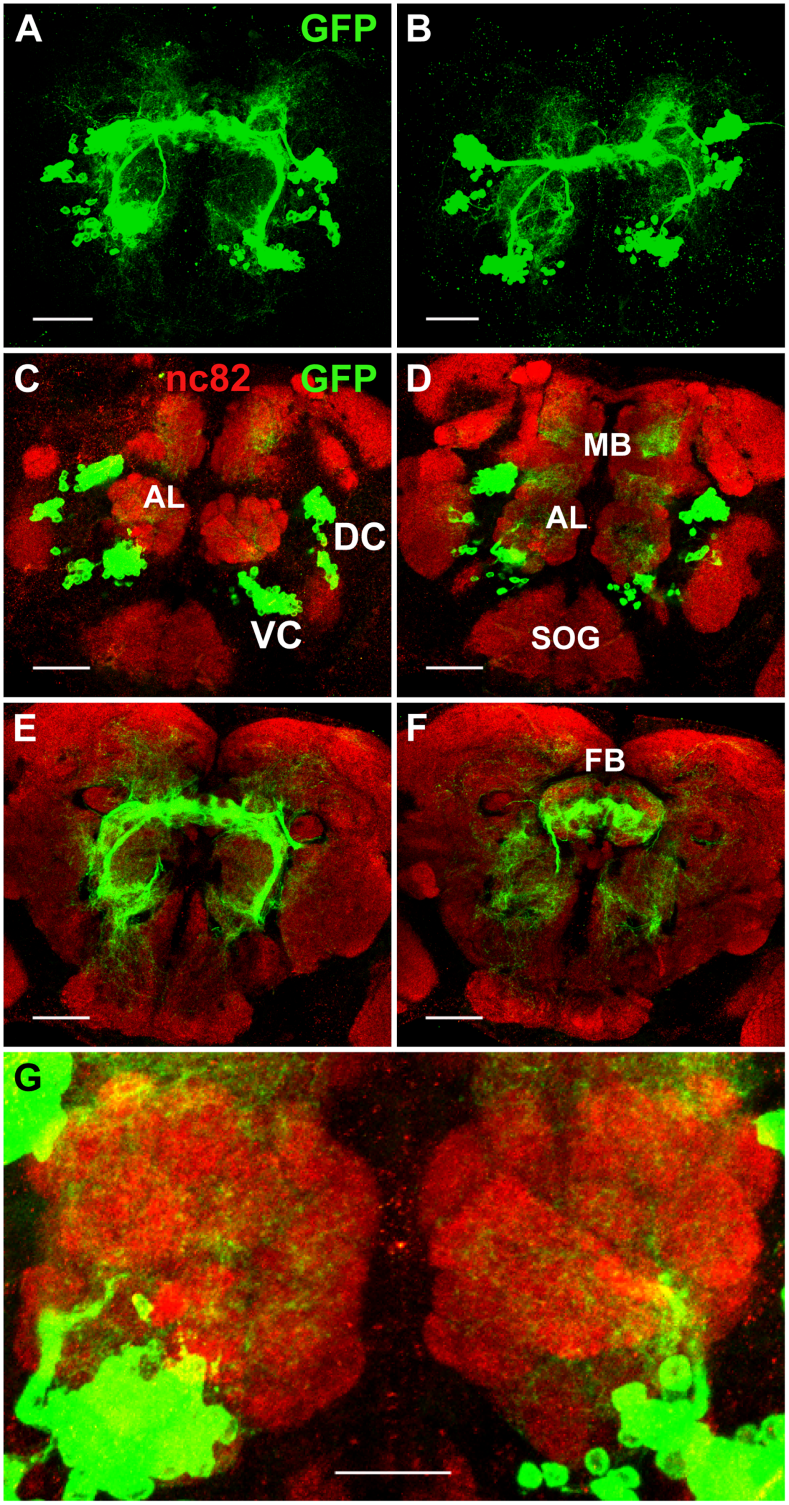
Projection patterns of *Poxn*-neurons in *Poxn* mutant adult brains. **(A,B**) Projections of *Poxn*-neurons in *w*^*1118*^; *Poxn*^*ΔM22-B5*^ *Poxn-Sbl-107*; *Poxn-CD8*::*GFP* (A) and *w*^*1118*^; *Poxn*^*ΔM22-B5*^; *Poxn-CD8*::*GFP* (B) *Poxn* mutant brains (dorsal side up), immunostained for GFP, show similar patterns at maximum intensity projection of Z-stacks extending over 85 μm (A) and 109 μm (B) at 40x magnification. (**C**–**F**) Substacks of (A), immunostained for GFP (green) and nc82 (red), are shown from anterior to posterior at 7–18 μm (C), 18–28 μm (D), 36–43 μm (E), and 43–49 μm (F). (**G**) Enlarged region of substacks of (A) at 0–31 μm, illustrating the reduced arborizations of *Poxn*-neurons that fail to invade the glomeruli of the ALs. AL, antennal lobe; DC, dorsal cluster; FB, fan-shaped body; MB, mushroom bodies; SOG, subesophageal ganglion; VC, ventral cluster. Scale bars: 50 μm (A–F) and 25 μm (G).

In adult brains of *Poxn* mutants (Fig 10B) or *Poxn* mutants rescued for all but the brain functions by *Poxn-Sbl* (Fig 10A), the projections of the *Poxn*-neurons are highly aberrant and fail to find the targets to which they connect in the wild type (Fig 9). The projections of the DC neurons do not shape the EB (Fig 10A and 10B) but form a degenerate structure, exhibiting a pattern of globules at the midline (Fig 10E and 10F), also apparent in paraffin sections of such brains (S9A’–S9D’ Fig). Frontal and horizontal paraffin sections of mutant brains demonstrate that the structure of the EB is formed neither in *Poxn* mutants (S9A’ Fig) nor in *Poxn* mutants rescued in all except the brain functions by *Poxn*-*Sbl* (S9B’–S9D’ Fig), but is replaced by several smaller globular structures. Moreover, as evident from the analysis of individual confocal layers, some projections aberrantly target the FB (Fig 10F), which is never observed in wild-type brains (Fig 9F).

The dendritic projections of the VC at the AL are dramatically reduced (Fig 10C, 10D and 10G) as compared to those in the wild type (Fig 9C, 9D and 9G), and no longer invade and innervate the glomeruli (Fig 10G). No mlALT is observed, and the LH is not targeted (Fig 10A and 10B). As already evident in mutant late third instar larvae (Fig 4G and 4H) and mutant pupae (Fig 8A'–8H'), the processes fasciculate and stall close to the dorsal *Poxn*-neurons, in the region where they would turn towards the LH in the wild type (compare Fig 10A, 10B and 10E with Fig 9A and 9F).

In *Poxn* mutant brains, their axons in some cases initially follow the mALT, like in wild type, but stall about at the location where in the wild type the mlALT leaves the mALT and bends dorsolaterally (Fig 6B). In other cases, they do not follow the mALT but follow the axons of the *Poxn*-neurons of the DC and stall together with these, i.e., before crossing the SEC (Fig 6C).

## Discussion

In this study, we have followed the fate of all cells that express Poxn in the developing brain of *Drosophila*. Poxn expression in the developing brain is restricted to postmitotic neurons, designated *Poxn*-neurons. Thus, no Poxn-expressing neuroblasts [41] or ganglion mother cells were observed. This contrasts with the developing peripheral nervous system where Poxn is expressed in sensory organ precursor cells and their derivatives [19,20,22,23,24,26]. The cell bodies of these *Poxn*-neurons occur in two bilaterally symmetrical clusters, a dorsal cluster, DC, in the protocerebrum and a ventral cluster, VC, in the deutocerebrum [19,42]. Their functions are restricted to the adult brain where the DC connects the bulb, BU, with the EB [10,14], and the VC links the glomeruli of the adult AL (i) with each other through LNs, and (ii) with the lateral horn, LH, by vPNs projecting their axons through the mediolateral antennal lobe tract, mlALT [15,16]. These connections depend completely on Poxn since in its absence the axons of *Poxn*-neurons do not extend properly and fail to find their targets. Thus, pathfinding of *Poxn*-neurons is impaired in *Poxn* mutants.

By the end of embryogenesis, a small number of *Poxn*-neurons expresses Poxn with bilateral symmetry, in a protocerebral DC of 20 neurons and a deutocerebral VC of 12 neurons. Only during third instar, larval *Poxn*-neurons begin to express Poxn until the number of *Poxn*-neurons reaches by late third instar about 242 in the DC and 109 in the VC (Table 1). Contrary to what might be expected, the embryonic *Poxn*-neurons have no larval functions, but some of them seem to form pioneer tracts, so-called primary axon tracts, PATs [62,63], during embryogenesis and first instar. The axons of the larval *Poxn*-neurons follow the PATs as secondary axon tracts (SATs) [62,64] during metamorphosis, as previously observed for many brain structures [57,65,66,67].

In addition to the developmental time of incipient Poxn expression, larval *Poxn*-neurons are distinguished from embryonic *Poxn*-neurons by their expression of Pros and absence of any overt differentiation of neurites. Moreover, embryonic *Poxn*-neurons can be identified in third instar larval as well as adult brains on the basis of their considerably larger size of nuclei and cell bodies compared to larval *Poxn*-neurons. Virtually all *Poxn*-neurons present at the end of larval development survive metamorphosis and no additional *Poxn*-neurons are formed. In the following, we discuss some implications of our findings in more detail.

### All *Poxn*-neurons are functional only in the adult brain

Considerable fractions of *Poxn*-neurons in the DC, 52%, and in the VC, 58%, are embryonic-born, as evident from the absence of BrdU incorporation into these *Poxn*-neurons after embryogenesis. However, none of these *Poxn*-neurons have a function in the larva, which is particularly surprising for the 12 and 20 embryonic *Poxn*-neurons of the VC and DC. Since all *Poxn*-neurons of the DC participate only in the formation of an adult brain structure, the EB, it is obvious that they have no larval function. For the embryonic and embryonic-born *Poxn*-neurons of the VC, the situation has to be examined more closely as one might expect them to innervate the larval AL. The following observations, however, argue against this possibility. First, no dendrites of *Poxn*-neurons are detectable that project to the larval AL (Fig 7; inspection of single layers of the confocal stack shown in Fig 5A). Second, postsynaptic Dlg is not expressed in *Poxn*-neurons at the larval AL (Fig 5A), nor is presynaptic Synapsin and Bruchpilot expressed at the LH during late third instar, where it would be expected if functional synapses had formed at these neuropils during embryogenesis. Third, the cell bodies of *Poxn*-neurons are located ventrally to the larval AL of third instar larvae, whereas those of larval PNs projecting to the larval AL are located anterodorsally and those of larval LNs ventrolaterally, laterally, or dorsally to the larval AL [31]. Fourth, in contrast to the vPNs expressing Poxn, which project directly to the LH and of which only four express *GH146*, most of the 21 larval PNs express *GH146*, all of which project via the MB to the LH [31,68,69]. These include some PNs born in the embryo that function in the larval AL and persist to innervate the adult AL [16,60]. We conclude that all *Poxn*-neurons possess only adult-specific functions.

### Embryonic and larval *Poxn*-neurons are primary and secondary neurons

The *Drosophila* brain originates from about 100 NBs, each with a unique identity, which delaminate in a stereotyped bilaterally symmetric pattern from the procephalic neurectoderm during stages 9–12 of embryogenesis [70,71,72]. These NBs divide during embryogenesis to give rise to about 100 bilaterally symmetric lineages of primary neurons and glial cells, which form the larval brain [62]. After a period of mitotic quiescence, the same NBs are reactivated to generate secondary lineages and secondary neurons [1,2,37,73,74] that terminally differentiate only during metamorphosis to form, together with remodeled surviving primary neurons, the adult brain [1,75]. Thus, it was thought that embryonic primary neurons build the larval brain, while larval secondary neurons, derived from the same NB, construct the adult brain. However, our findings do not fit this scheme for two reasons: (i) many embryonic-born *Poxn*-neurons terminally differentiate only during metamorphosis to form adult brain structures, and (ii) the embryonic primary *Poxn*-neurons do not participate in the formation of the larval brain, only in that of the adult brain. Yet, consistent with our results, it was found more recently that terminal differentiation of late embryonic-born neurons can be arrested until metamorphosis [76]. Such neurons behave like secondary neurons, and hence were called embryonic-born secondary neurons [76]. Accordingly, it was proposed to call them simply secondary neurons, classifying primary versus secondary neurons in general on the basis of their time of differentiation rather than of birth [76,77], a suggestion which we follow here. Hence, what we called embryonic and larval *Poxn*-neurons correspond to primary and secondary neurons, respectively. In this context, it is interesting that NBs of the AL and MB have been reported much earlier to proliferate continuously during the transition from late embryonic to early postembryonic stages [2]. Our results further support the early notion that apparently fixed lineages of NBs may dictate that some adult neurons are generated by embryonic divisions although these neurons do not terminally differentiate until metamorphosis [37].

Two features, in addition to their time of terminal differentiation, distinguish embryonic primary *Poxn*-neurons from embryonic-born secondary *Poxn*-neurons. First, like all primary neurons [48,50,51], primary *Poxn*-neurons do not express Pros, whereas embryonic-born secondary *Poxn*-neurons do, as do larval-born secondary neurons [49,78]. Since Pros is no longer expressed in terminally differentiating primary neurons [48,51], we might speculate that Pros is also downregulated when terminal differentiation of secondary neurons occurs during metamorphosis. If true, this would indicate that Pros not only promotes cell cycle exit in the GMC precursors of primary neurons [47] and immature secondary neurons, but also inhibits terminal differentiation in the latter before metamorphosis. Second, the cell bodies or nuclei of embryonic primary *Poxn*-neurons are strikingly larger and located more deeply in the cortex (S7 Fig), and are less tightly packed (S5 Fig) than those of secondary or larval *Poxn*-neurons, in agreement with earlier reports [52,63]. This switch from larger primary neurons, located in deeper layers, to smaller embryonic-born and larval secondary neurons, observed in more superficial layers, is achieved by a transition from Chinmo [79] to Br-Z3 expression regulated by Cas and Svp [52,76].

### Most vPNs of the adult antennal lobe are *Poxn*-neurons

Many *Poxn*-neurons of the VC project their axons from the AL to the LH and hence are so-called projection neurons (PNs) that connect the input received from olfactory sensory neurons (OSNs) to a higher brain center, the LH [15,16,17,18,27,28,80,81,82]. Since the axons of these *Poxn*-neurons bypass the MB and directly connect the AL with the LH through the mlALT, they are vPNs, which is consistent with the location of their cell bodies immediately ventral to the AL. vPNs are derived from a ventral neuroblast (vNB) and can be marked by the expression of the GAL4 enhancer trap lines *MZ699* [17,82] or *GH146* [16,28]. Postembryonic induction of vNB clones in first instar larvae within 2 hours after hatching has demonstrated that these consist of 49±6 vPNs, which are marked either by *MZ699* or *GH146*, but not both, and ipsilaterally project through the mlALT directly to the LH [15,17,18,82]. There are 7 *GH146*-labeled neurons in clones induced in early embryos [80] (S1 Table and S10A Fig), which are observed also in clones induced in newly hatched larvae [18]. In addition, there are 45±5 (s.d.) vPNs that express *MZ699*-GAL4 in clones derived from the postembryonic vNB [18].

We found that 46 (42%) of the 109 *Poxn*-neurons of the VC (Table 1) are labeled by BrdU and thus would be observed in postembryonic clones, only 4 of which express *GH146*, while 3 *GH146*-positive vPNs do not express Poxn (S1 Table and S10A Fig). Thus, there are 42 Poxn^+^ GH146^-^, 4 Poxn^+^ GH146^+^, and 3 Poxn^-^GH146^+^ vPNs, which add up to 49 postembryonic vPNs. Assuming an upper limit of 55 vPNs in postembryonically induced clones marked by either *GH146* or *MZ699* [18], we estimate that there are at most 48 MZ699^+^ vPNs, 42 of which would be Poxn^+^ and 6 Poxn^-^. Since the 4 *Poxn*-neurons that express *GH146*, do not express *MZ699*, 42/46 (91%) postembryonic *Poxn*-neurons express *MZ699*, which is consistent with our observation that most *Poxn*-neurons of the VC express *MZ699*, namely 93±2% or 101 (S1 Table and S10B Fig). We conclude that a similar fraction of embryonic-born *Poxn*-neurons express *MZ699*. Finally, this result is entirely consistent with our assumption that all 46 *Poxn*-neurons of clones induced after larval hatching are vPNs projecting through the mlALT. Since there are at most 9 postembryonic vPNs that do not express Poxn, 3 expressing *GH146* and at most 6 expressing *MZ699*, most but not all vPNs of the AL express Poxn.

In addition to the 49±6 vPNs of postembryonic origin, 46 of which are *Poxn*-neurons, our observation that *Poxn*-neurons extend their axons through the mlALT already in first instar larvae (Fig 4A) argues that up to 12 additional primary *Poxn*-neurons project through the mlALT and hence are vPNs. Thus, up to (46+12)/(49+12), i.e., 95% of the vPNs of the AL are *Poxn*-neurons.

### *Poxn*-neurons of the VC that are not vPNs are local interneurons derived from embryonic NB(s)

Since 58% of the 109 *Poxn*-neurons in the VC (Table 1) are not labeled by BrdU, 63 *Poxn*-neurons are derived from embryonic rather than postembryonic vNBs. These embryonic-born *Poxn*-neurons include, in addition to the 12 embryonic *Poxn*-neurons, 48±7% (s.d.) of all *Poxn*-neurons (or 52±8 *Poxn*-neurons) that are labeled by the enhancer trap marker *KL107* (S1 Table and S10C Fig). Therefore, most if not all *Poxn*-neurons labeled by *KL107* are derived from embryonic NB(s). It is known that the enhancer trap markers *KL107* and *GH298* label exclusively local interneurons (LNs) in postembryonic clones of the lateral NB lineage of AL neurons [18,83]. None of the *Poxn*-neurons is labeled by *GH298* (S1 Table). If we assume that *KL107* exclusively labels LNs also in embryonic clones, 52±8 *Poxn*-neurons derived from embryonic vNB(s) would be LNs.

Two observations argue that *Poxn*-neurons expressing *KL107* are not derived from lateral but rather from ventral embryonic NB(s). First, all cell bodies of the *Poxn*-neurons are located ventrally to the most ventral neurons of the lAL lineage. These express *acj6-GAL4* [18], which is not expressed in *Poxn*-neurons (S1 Table), in agreement with the observation that *acj6-GAL4* is not detectable in vPNs [18]. Second, *Poxn*-neurons expressing *KL107* are located ventral to many *Poxn*-neurons that are not labeled by *KL107* and are vPNs (S10C Fig). Therefore, the 52±8 *Poxn*-neurons that express *KL107* are derived from NB(s) during embryogenesis. It was reported that there are 104±9 ventral LNs in clones induced by MARCM throughout development and marked by the GAL4 line *OK107* (not the same as *KL107*), which thus include embryonic clones derived from vNB(s) [84]. These vLNs consist of 54±13 GABAergic and 59±9 glutamatergic neurons [84]. Some or all of the *Poxn*-neurons that express *KL107* may correspond to a subset of these GABAergic and glutamatergic vLNs, most of which project bilaterally to only few glomeruli with symmetrical glomerular innervation patterns in ipsilateral and contralateral ALs [84]. Alternatively, most of these *KL107 Poxn*-neurons correspond to hitherto uncharacterized vLNs derived from embryonic vNB(s). Unidentified NB lineages that include LNs of the AL are thought to be likely to exist [85]. To derive some 50 *Poxn*-neurons as well as the 12 primary *Poxn*-neurons of the VC from a single embryonic Type I NB would require 31 asymmetric NB divisions, each of which lasts at least 45 min [2,86]. Thus, if divisions begin at 3.75 hours of development at 25°C (stage 9 of embryogenesis), divisions of a single embryonic NB to generate 62 embryonic-born neurons would end at 27 hours of development. Since larvae labeled by BrdU hatched on BrdU-containing food, BrdU-incorporation is expected to be detected within 2–3 hours after hatching, i.e., at about 24–25 hours of development at 25°C. Therefore, at least two Type I NBs are required to produce the 62 embryonic-born *Poxn*-neurons of the AL. As NBs of the AL may proliferate continuously during the transition from late embryonic to early postembryonic stages [2], one might explain the 50 embryonic-born vLNs to be derived from one NB and the 12 primary *Poxn*-neurons from another. If embryonic-born neurons of the ventral AL lineage are derived from a hemilineage, like the postembryonic adPN and vPN lineages of the AL [87], the 12 primary *Poxn*-neurons might be derived from a single embryonic vNB after 12 divisions, i.e., by 14 h of development at 25°C or stage 16 of embryogenesis, which would be consistent with our observations (Table 1).

An important consequence of all these considerations is that virtually all *Poxn*-neurons that are vLNs are BrdU(–) and hence go through their last S-phase by the end of embryogenesis. Indeed, they pass even through their last mitosis during embryogenesis because they are not included in postembryonic clones, which consist of 49±6 vPNs [18]. By contrast, the *Poxn*-neurons that are vPNs are derived from the larval vNB with the exception of some or all of the 12 embryonic primary *Poxn*-neurons. Interestingly, for the lateral lineage it has been reported that PNs are generated during later divisions than LNs [88]. Our results may suggest this to be a general characteristics of LNs and PNs of the AL.

### *Poxn*-neurons are large-field R neurons of the ellipsoid body

The EB neuropil is part of the CX [8] and has been shown to consist largely of small-field neurons, which interconnect the neuropils of the CX, and large-field neurons, which connect the EB with neuropils outside of the CX [10,14]. The toroidal structure of the EB neuropil has been divided along the anteroposterior axis into rings [10] or, more recently, three shells [89], while concentric rings along the radius are called layers [61,89]. Finally, 16 sectors [10], or wedges (and demi-wedges), which segment the toroid and are input domains, and eight tiles, which are output domains, have been discovered [89]. The large-field neurons, the cell bodies of which occur in two bilaterally symmetric clusters, form so-called ring neurons, transferring information from the ipsilateral BU to the EB through the lateral EB tract [10,61]. They are subdivided into several classes R1–R3, R4-distal (R4d), and R4-medial (R4m) according to their patterns of arborization at the EB [10,14,61]. Characterization of these neurons by Golgi staining permitted the classification of single neurons into the classes R1–R4 arborizing in concentric layers [10], while staining of neurons with suitable GAL4 enhancer trap lines allowed a subdivision of R4 into R4d and R4m [14]. R1–R3 neurons project to the EB through the central EB canal and arborize outwardly in different layers, while R4 neurons reach the periphery of the EB and arborize inwardly in the outer layer, either distally (R4d) or more medially (R4m).

Our staining of the EB by the expression of Poxn cannot distinguish between different classes of ring neurons. However, analysis of the overlap of *Poxn*-neurons of the DC with the expression of the GAL4 enhancer trap lines used by Renn et al. [14] showed that all R-sublines—c105/c561 (R1), c42 (R2, R4m), c547/c819 (R2, R4m), c232 (R3, R4d), and 189Y (R3)–labeled relatively small subsets of the *Poxn*-neurons (F. Schmid, WB, MN, unpublished results). For line 189Y, where GAL4 was thought to be inserted in the *foraging* gene at 24A2-4, we found GAL4 had actually inserted in the *lilliputian* gene at 23C and hence called this line *P{GawB}lilli* (F. Schmid, WB, MN, unpublished results). That these lines labeled subsets of *Poxn*-neurons is consistent with our observation that their expression is absent in *Poxn* mutants and thus at least indirectly depends on Poxn (F. Schmid, WB, MN, unpublished results). Although this shows that *Poxn*-neurons of the DC represent all classes of R neurons [90], these enhancer trap lines include at most about half of all *Poxn*-neurons of the DC (our unpublished results). Since our results do not show any *Poxn*-neurons that do not connect the BUs with the EB, we conclude that these lines do not cover all neurons forming the lateral EB tract, in agreement with the estimate that there are over 200 neurons in the R1–4 group [61]. Indeed, there is no evidence that the about 242 *Poxn*-neurons of the DC (Table 1) do not constitute all neurons that connect the BUs with the EB. Our results at least indicate that they are the overwhelming majority of the neurons forming the lateral EB tract. In agreement with this conclusion, we observe no EB in *Poxn* mutants (S9 Fig). Therefore, expression under the control of *Poxn* may serve as an important new marker that specifically labels most, if not all, large-field R neurons of the developing and mature EB.

### Are embryonic *Poxn*-neurons pioneering neurons?

It has been shown that the ventral fascicle of the SEC tract is a pioneer tract formed by two bilaterally symmetrical P2l clusters of neurons during stages 13 to 15 of embryogenesis [65,67]. This P2l neuropil founder cluster probably corresponds to some of the *Poxn*-neurons of the DC that extend axons into the SEC and meet at the midline as early as at stage 14 of embryogenesis (S3 Fig). This meeting of their axons are the earliest sign of the future adult neuropil structure of the EB. The axons of the other primary *Poxn*-neurons of the DC follow the paths of these early *Poxn*-neurons to form a PAT [62,63]. During metamorphosis, the remaining 222 *Poxn*-neurons of the DC probably use these PATs as guide to form the SATs [62,64] and the EB neuropil. It is possible, however, that the lateral EB tract observed in the late pupal or adult brain (Figs 8 and 9) [10,14] consists of several tracts at larval stages all of which have their PATs. Thus one might speculate that the four tracts formed by embryonic primary *Poxn*-neurons at late third instar (Fig 5B) may correspond to pioneering tracts for R1–R4. The three ventral tracts could correspond to R1–R3 that will arborize outwardly from the EB canal, while the dorsal tract might be R4 that will arborize inwardly from the periphery of the EB [10,14]. Perhaps even five tracts leading from the DC to the midline can be distinguished in third instar brains (Fig 6E–6H), which might correspond to the five classes of R neurons, R1-R3, R4d, and R4m [14].

Although pioneer tracts have been observed for the antennocerebral tract (ACT) that connects the AL via the MB to the LH at the end of embryogenesis [57,65,67], no pioneer tracts have been described for the mlALT, which directly connects the AL with the LH, bypassing the MB, and is formed by vPNs of the AL, most of which are *Poxn*-neurons. However, in first instar larvae embryonic primary *Poxn*-neurons of the VC exhibit a tract that may be such a pioneer tract of the mlALT (Fig 4A), which persists throughout larval development (Fig 4B and 4C). Consistent with such a role, this tract is followed by SATs of all secondary vPNs of the AL during metamorphosis (Fig 8A–8H), most of which are secondary *Poxn*-neurons as argued above. By contrast, the few vPNs that are negative for Poxn but express *GH146* or *MZ699*, are all postembryonic [18] and hence unlikely to be pioneering neurons of the mlALT.

### *Poxn*-neurons are major parts of lineages forming the EB and AL

It was first recognized by Ito et al. [91] through lineage analysis that the clonal units of brain NBs form the structural units of the adult brain, such as axon tracts and neuropil compartments [66,77]. This concept has been confirmed by numerous subsequent studies [18,52,62,63,64,67,74,85,88,92,93,94,95,96,97] and is also entirely consistent with our analysis of the development of *Poxn*-neurons. Thus, we find that (i) all *Poxn*-neurons of the DC are part of the adult brain structure connecting the lateral bulb with the EB through the lateral EB tract [10,14], and (ii) all *Poxn*-neurons of the VC are part of the adult brain structure connecting the AL with itself and directly to the LH through the mlALT, thus by-passing the MB. A simple comparison shows that *Poxn*-neurons of the DC and VC are part of the *per*-positive lineages DALv2 and BAla1 [63,74,98], respectively.

Although no lineage analysis was performed for *Poxn*-neurons, we may estimate how many NBs are necessary to generate the *Poxn*-neurons of the DC and VC lineages. On the basis of a cell cycle time of 1.1–1.5 hours, it had been estimated that Type I NBs, after resuming their activity by mid-larval development, may undergo 40–70 additional mitoses to generate secondary neurons [2,99]. To produce 116 BrdU^+^ *Poxn*-neurons of the DC, an NB would have to divide about 60 times. Therefore, this number of *Poxn*-neurons could be derived from a single NB if the *Poxn*-neurons of the DC are not part of a hemilineage like that of the vPNs of the AL [87]. However, in addition to the 116 BrdU^+^ *Poxn*-neurons, 126 BrdU^-^*Poxn*-neurons have to be derived from the same embryonic NBs. As reasoned above, this would require 63 divisions or about 47 hours of development at 25°C [2,86]. Hence, to generate the 126 primary and embryonic-born secondary *Poxn*-neurons at least 2 NBs are required if we assume a cell-cycle time of 45 min and that these NBs continue to divide for about 3 hours after embryogenesis. Two NBs, however, would also suffice to produce 116 larval-born secondary *Poxn*-neurons, even if they are part of a hemilineage. Thus, at least 2 NBs are required to generate all *Poxn*-neurons that are part of what has been described as DALv2 lineage, which gives rise to the R neurons of the EB [63]. Indeed, we have no evidence that neurons different from the *Poxn*-neurons of the DC participate in the formation of the lateral EB tract of R neurons [10,14]. Thus, these *Poxn*-neurons may well form entire lineages derived from two NBs per brain hemisphere. This conclusion is consistent with our observation that in the absence of Poxn the EB does not form properly (Fig 8A’–8H’) although a distorted neuropil is observed in adult brains (Fig 10 and S9 Fig). The latter observation is not surprising, if we consider that many neurons that presynaptically attach to the PB may still attempt to connect their axons postsynaptically at the location where the EB neuropil would reside [89]. Our comparison with the DALv2 lineage further suggests that this lineage is based on two rather than a single NB.

In the case of *Poxn*-neurons of the VC, we argued above that at least two embryonic Type I NBs are required per brain hemisphere to generate the 12 primary and 51 embryonic-born secondary *Poxn*-neurons of the AL. To account for the 46 BrdU^+^ larval *Poxn*-neurons, it is easy to see that one larval NB, the vNB [18,74], is sufficient, even though this ventral BAla1 lineage [18,63,74] is a hemilineage of vPNs [87] and includes a few additional Poxn^-^neurons labeled by MZ699 or GH146 (s. above). Since there are two types of *Poxn*-neurons, vPNs and vLNs, one NB may generate all vPNs, the other all vLNs. Thus, one NB may first produce all primary vPNs in a primary lineage and subsequently, after embryogenesis, all secondary vPNs in the secondary BAla1 lineage. Since no clones of vLNs induced after embryogenesis have been observed [18,63,84], the other NB may produce all vLNs during embryogenesis.

### Poxn regulates axon pathfinding and dendritic targeting

In *Poxn* mutants, *Poxn*-neurons of the DC and VC do neither find their targets postsynaptically at the BU and AL nor presynaptically at EB and LH, respectively. Accordingly, these neuropils are not properly formed (Figs 8 and 10, S9 Fig). It follows that in the absence of Poxn dendritic targeting and axon pathfinding are impaired in these neurons. Therefore, Poxn plays a decisive role in the control of these processes. This regulation may occur indirectly and/or directly. If regulation is indirect, possible targets in the VC are *cut* and/or *lim1*, which themselves encode transcription factors expressed in vPNs, in the absence of which dendritic targeting to glomeruli of the AL is impaired in vPNs [100]. However, *cut* as target of Poxn seems unlikely because Cut is already expressed in the vNB, i.e., before Poxn, and *cut* rather controls dendritic targeting in all PNs in a global manner [100]. Rather it seems that, like in p-es organs of the PNS, Poxn acts in combination with Cut [22]. Although the *lim1* gene might be a target of Poxn, the targeting defect in *lim1* mutants is limited to uniglomerular vPNs that target DA1 [100] and hence does not explain the drastic targeting defects of vPNs in the AL of *Poxn* mutants. The combinatorial model of transcription factors regulating target specificity [100], in agreement with the early observation that PN targeting in the AL is specified by lineage and birth order [28], is more consistent with our results for Poxn. However, in *Poxn* mutants targeting is not only defect in vPNs but also in LNs and not only in the AL but also in the LH. In addition, all *Poxn*-neurons of the DC fail to target the BU and EB in *Poxn*-mutants. These findings argue for a general targeting defect in the absence of Poxn in these neurons. A similar general targeting defect of adPNs was observed in the absence of the POU domain transcription factor Acj6, which is specifically expressed in adPNs [40].

A direct target of Poxn might be the *baz* gene, encoding a member of the Par complex since the phenotypes of *baz* null mutant neurons of the *per*-positive post-embryonic lineages BAla1 and DALv2 [98], are similar to, though weaker than, those of *Poxn* mutants (Figs 6B, 6C and 10). Consistent with this possibility, Bozooka is re-expressed in postmitotic neurons at the third larval instar. Since the formation of dendrites at the BU and AL is clearly impaired in the DC and VC of *Poxn* mutants (Figs 8 and 10G), another possible target gene of Poxn shown to be required for proper elaboration of dendritic fields at least in GH146-positive vPNs is *Dscam* [101]. Also in this case, however, the *Poxn* mutant phenotype is much stronger.

Clearly, axon guidance of *Poxn*-neurons is impaired in the absence of Poxn, as their axons do not reach their targets at the EB and LH (Figs 8 and 10). It is possible that this axon guidance defect results from the absence of Poxn in pioneering primary *Poxn*-neurons that form PATs, while secondary *Poxn*-neurons may not need Poxn to follow the PATs. This possibility is supported by the fact that secondary *Poxn*-neurons follow aberrant tracts in *Poxn* mutants (Figs 4E–4H, 5C, 5D, 6B, 6C and 8A’–8H’) until their axons are stalled.

### Poxn does not determine the synthesis of neurotransmitters in the DC

The BU, EB, and both tracts of the large-field R neurons, the RF-tract running from their cell bodies to the BU and the lateral EB tract leading from the BU to the EB, strongly stain for the neurotransmitter GABA [10]. Since most, if not all, of these neurons are *Poxn*-neurons, one might conclude that most *Poxn*-neurons of the DC are GABAergic and thus inhibitory. However, this conclusion is premature, as shown recently for R neurons [90]. Based on a number of GAL4 enhancer trap lines expressed in R neurons [14], it was shown that the neurotransmitters GABA, acetylcholine, and dopamine are each synthesized by different sets of R neurons [90].

Similarly, it was found that most MZ699^+^ vPNs of the AL express the GABA synthesis enzyme Gad1 [82]. Strikingly, all of the ~ 90 adPNs and lPNs expressing *GH146* are GABA-negative, whereas all of the few GH146-positive vPNs are GABA-positive [102,103]. Thus, although most PNs of the AL are not GABAergic, most of the vPNs are [102]. Therefore, again most, if not all, *Poxn*-neurons that are vPNs are GABAergic. For the ~ 50 embryonic-born *Poxn*-neurons that are probably ipsilateral LNs (s. above), the situation is not clear. Although most LNs of the AL, identified by various enhancer trap lines, are GABAergic [82,102], we cannot assign any of these lines to the ~ 50 *Poxn*-LNs [84]. Even if these *Poxn*-LNs are a subset of the vLNs expressing the GAL4 enhancer trap line *OK107* [84], it is possible that many of these vLNs use glutamate rather than GABA as neurotransmitter (s. above). Thus, although it is conceivable that all *Poxn*-neurons of the VC are GABAergic, many Poxn-neurons of the DC are not. Therefore, at least in the DC Poxn does not determine the synthesis of neurotransmitters.

### Ellipsoid body formation in *Drosophila* may reflect phylogeny

The protocerebrum of most insects and collembolans includes a small number of prominent interconnected midline neuropils with similar structures, called CX [10,11,104]. The neuropils of the CX, particularly also the EB [105], have been shown to be crucial for the coordinate control of locomotor activity and orientation behavior in adult flies [106,107,108]. In addition, the ring neurons of the EB mediate in a redundant fashion a short-term spatial orientation memory during locomotion, perhaps similar to the working memory in vertebrates [109,110,111]. It has been suggested that an increasing complexity of the architecture of CX neuropils in insects correlates with a more sophisticated limb motor repertoire [11,12]. In most insects, the EB forms an arch-like neuropil of distinct layers, but in *Drosophila* this arch is extended into a complete torus [11,112]. Accordingly, it has been postulated that there is a plausible phylogenetic relationship among neopteran insects with regard to ellipsoid body formation [11].

In the late larval and early pupal brain of *Drosophila*, the primordial EB consists of commissural tracts (Figs 5B, 6B and 8A) and resembles the EB of the adult silverfish *Lepisma saccharina*, a small wingless insect [12]. By 30 hours APF, the projections of the *Poxn*-neurons of the DC, which eventually form the EB, assume the distinct shape of an arch-like or kidney-shaped structure (Fig 8E), similar to the EB of the adult littoral earwig *Anisolabis maritima* [12], the desert locust *Schistocerca gregaria* [113], or the dragonfly *Pachydiplax longipennis* [114]. By 45 to 50 hours APF, the projections of the large-field R-*Poxn*-neurons of the developing EB give rise to the doughnut-like adult form of the EB (Fig 8G and 8H). These transient structures of the EB during metamorphosis may thus be a relict of how the EB evolved in dipterans while in other insects it remained kidney-shaped [104,115]. Interestingly, in the *ellipsoid body open* mutant *ebo*^*KS263*^ the ventral region of the EB is not completely closed [106,107]. It thus resembles the wild-type EB at 40 hours APF (Fig 8F) and suggests that the EB defect in *ebo*^*KS263*^ mutants occurs at this specific stage of development.

The toroidal form of the EB in the adult *Drosophila* appears to correlate with a considerable increase in number of neurons that give rise to the lateral EB tract. While in the desert locust these tracts are formed by about 100 bilateral pairs of neurons [113], in *Drosophila* this number is more than twice as large (Table 1). Thus, the extension of the arch-like form of the EB, observed in insects like the locust, cockroach, or dragonfly, to a toroidal EB in dipterans like *Drosophila* has not occurred without the recruitment of many additional R-neurons during evolution. This increase in the number of neurons, however, was independent of *Poxn* activity, as *Poxn* is expressed only in post-mitotic neurons. The observation of a more elaborate EB in more complex insects is in line with the suggestion that a higher complexity of the CX neuropils correlates with a larger repertoire of locomotion control [11,12].

## Supporting information

S1 Table Coexpression of GAL4 lines with Poxn in the ventral cluster of the adult brain. The fractions of *Poxn*-neurons expressing the GAL4 enhancer trap lines in the VC of the adult brain were determined by examining their coexpression with the enhancer trap lines in single layers of CLSM sections, examples of which are shown as maximum intensity projections in S10 Fig. Standard deviations were determined from four ventral clusters each. The overlap of Poxn expression with that of the enhancer trap line *GH146-GAL4* was constant. There was no coexpression of Poxn with *GH298-GAL4* and *acj6-GAL4*.
(TIF)

S1 Fig Map of *Poxn* transgenes. Maps of the *Poxn* constructs that rescue as transgenes all *Poxn* functions, *Poxn-SuperA*, or all but the *Poxn* brain function, *Poxn-Sbl*, and of the *Poxn*-driver, *Poxn-Gal4-13*, and *Poxn*-reporter construct, *Poxn-CD8*::*GFP*, are shown with regard to a restriction map of the *Poxn* locus (numbers in parentheses refer to distances in base pairs from the ‘upstream’ transcriptional start site S; cf. [19]). *Poxn-Gal4-13* [19] and *Poxn-CD8*::*GFP* express Gal4 and the CD8::GFP fusion protein, respectively, under the control of the *Poxn* upstream enhancers. The composition of the *Poxn* constructs is illustrated in colors representing the *Poxn* upstream region (light green), 5’- and 3’-UTRs and out-of-frame *Poxn* coding region (dark orange), introns (light orange), coding region (black), and downstream region (light blue), and the coding regions of Gal4 (magenta), CD8 (gray), and GFP (dark green). The orange and black arrows indicate start and direction of transcription and translation, respectively. Restriction sites: Ag, *Age*I; As, *Asc*I; B, *Bam*HI; E, *Eco*RI; H, *Hin*dIII; N, *Not*I.
(TIF)

S2 Fig *Poxn*-neurons in first and second instar larval brains. **(A)** The *Poxn*-nuclei in one hemisphere of an *Ore-R* late first instar larval brain (45 h AEL; Table 1), stained for Poxn (green) and Elav (red), are shown in an Imaris surpass view of the entire Z-stack extending over 43 μm at 63x magnification (same image as in Fig 2A). Only the green channel is shown. The *Poxn*-nuclei of the VC and DC are numbered from anterior to posterior, and all of these in the VC are anterior to those in the DC. Thus, *Poxn*-nuclei 14 and 15 of the DC are superimposed but posterior to *Poxn*-nuclei 6 and 5, respectively, as evident from inspection of individual layers of the Z-stack. Similarly, analysis of single layers showed that all *Poxn*-nuclei are positive for Elav, whereas some smaller green spots do not stain for Elav (not shown). (**B)** The *Poxn*-nuclei of a *w*^*1118*^; *Poxn-CD8*::*GFP* late second instar larval brain (68 h AEL; Table 1), stained for Poxn (red) and GFP (green), are shown as a maximum intensity projection of CLSM sections of a Z-stack extending over 50 μm at 20x magnification. Numbering of the cell bodies of *Poxn*-neurons in the DC and VC of the right and left hemispheres, shown in the upper and lower part of the image, is from anterior to posterior. The brain is the same as that shown in Fig 4B where only the green channel is shown. Scale bars: 10 μm (A) and 20 μm (B).
(TIF)

S3 Fig *Poxn*-neurons of the dorsal cluster differentiate neurites in the embryonic brain by stage 14. **(A,B)** *Poxn*-neurons in the brain of a *w*^*1118*^; *Poxn-Gal4-13-1 UAS-GFP/TM6B* stage 14 embryo are visualized by immunofluorescent staining for GFP (green) and Elav (red), and are analyzed in both channels (A) or only the green channel (B). Panels show an Imaris surpass view of a substack (that eliminates some cell bodies but shows all projections of *Poxn*-neurons) of a Z-stack extending over 42 μm at 63x magnification with anterior up. Inspection of single confocal layers reveals that all Poxn-expressing cells in the brain also express Elav and hence are post-mitotic neurons. While the *Poxn*-neurons of the VC do not yet show overt signs of neurite differentiation, the *Poxn*-neurons of the DC clearly differentiate neurites and extend axons along several tracts that appear to meet at the midline in the SEC (white arrowheads). Scale bar: 20 μm.
(TIF)

S4 Fig None of the Poxn-expressing cells are glia. Cells expressing Poxn (green) and Repo (red) are visualized by immunofluorescent staining of the brain of a *w*^*1118*^; *Poxn-CD8*::*GFP* late third instar larva. The brain region shows a DC and VC as maximum intensity projection (of a Z-stack extending over 35 μm) along the A/P axis at 63x magnification. No colocalization of GFP and Repo is observed, which would be revealed as white pixels. Neurites are not visible because the image was taken at low exposure for optimal visibility of cell bodies. Scale bar: 10 μm.
(TIF)

S5 Fig *Poxn*-neurons of late third instar larval brains that express Poxn only after embryogenesis express Pros and show no outgrowth of neurites. (**A**–**C**) The *Poxn*-nuclei in one hemisphere of an *Ore-R* late third instar larval brain, stained for Poxn (red) and Pros (green), are shown as 1 μm sections at 9–10 μm (A), 15–16 μm (B), and 21–22 μm (C) of a confocal Z-stack extending from 0 (anterior) to 36 μm (posterior). Note that all *Poxn*-nuclei stain for Pros except 20 of the DC and 12 of the VC, some of which are visible in panels B (4 and 3 in DC and VC, respectively) and C (2 and 3 in DC and VC, respectively), as indicated by arrowheads. These nuclei, which do not stain for Pros, are at the large end of the size distribution of *Poxn*-nuclei (S7A and S7B Fig), show the by far highest levels of Poxn protein, and are the same as those that stain for Poxn in late embryos or in first and second instar larvae (S2 Fig and Table 1). (**D**–**F**) The *Poxn*-neurons in one hemisphere of a *w*^*1118*^; *Poxn-CD8*::*GFP* late third instar larval brain, stained for Pros (red) and GFP (green), are shown as 1 μm sections at 16–17 μm (D), 22–23 μm (E), and 27–28 μm (F) of a confocal Z-stack extending from 0 (anterior) to 50 μm (posterior). A maximum intensity projection of the entire Z-stack in the green channel is shown in S6 Fig to which the numbers of *Poxn*-neurons that do not express Pros refer. These reveal neurites, in contrast to *Poxn*-neurons that express Pros, which show no overt signs of differentiation. All images were taken at 63x magnification. Scale bars: 10 μm (A–C) and 5 μm (D–F).
(TIF)

S6 Fig The numbers of *Poxn*-neurons in the DC and VC of late third instar larval brains that do not express Pros correspond to those of the embryonic *Poxn*-neurons. The *Poxn*-neurons in one hemisphere of a *w*^*1118*^; *Poxn-CD8*::*GFP* late third instar larval brain, stained for Pros (red; not shown) and GFP (green), are shown as an Imaris surpass view of the entire Z-stack extending over 51 μm at 63x magnification. Only the green channel is shown. The confocal picture is the same Z-stack of which three sections are shown in S5D–S5F Fig The *Poxn*-neurons in the DC and VC that do not express Pros are numbered from anterior to posterior (or ventral to dorsal of the flattened CNS), and all of these in the VC are anterior to those in the DC. Their numbers, 20 in the DC and 12 in the VC, correspond to those of the embryonic *Poxn*-neurons present by late embryogenesis and in first and second instar larvae (Table 1; S2 Fig). These embryonic *Poxn*-neurons of late third instar larvae also exhibit the highest levels of CD8::GFP, which is consistent with the highest levels of Poxn protein observed in the nuclei of these *Poxn*-neurons (S5A–S5C Fig). Scale bar: 10 μm.
(TIF)

S7 Fig **(A,B) Embryonic *Poxn*-neurons of late third instar larvae differ from larval *Poxn*-neurons by the absence of Pros and their larger nuclear size.** The nuclei of the *Poxn*-neurons in one hemisphere of a late third instar *Ore-R* brain, three sections of which are shown in S5A–S5C Fig, were numbered (Table 1), and their diameters measured and averaged over three orthogonal sections. The histograms for the dorsal (A) and ventral clusters (B) show the distributions of the nuclear size classes that are indicated in panels C and D for *Poxn*-nuclei labeled (green) and not labeled by Pros (red). Note that there are 20 (DC) and 12 (VC) *Poxn*-nuclei that lack Pros, which equals the number of embryonic *Poxn*-neurons (Table 1), and that these have the largest nuclei. The largest *Poxn*-neurons have an average nuclear diameter of 3.4 ± 0.15 μm (s.d.; 7 of the arbitrarily chosen units shown in the histograms correspond to 3.5 μm). **(C,D) Skewed nuclear size distribution of *Poxn*-neurons along the anteroposterior axis in the late third instar brain**. The distributions of the size classes of *Poxn*-nuclei, shown in panels A (C) and B (D), are shown along the anteroposterior axis with respect to the Z-stack intervals indicated on the abscissa. In both the DC (C) and VC (D), there is a striking bias in the nuclear size distribution with larger nuclear diameters closer to the posterior. When plotted, the average nuclear diameters increase roughly in a linear fashion with increasing distance from the anterior. No distinction was made between nuclei displaying high, medium, and low levels of Pros or no Pros, classes that can be easily distinguished in sections (S5A–S5C Fig).
(TIF)

S8 Fig **(A,B) The largest *Poxn*-nuclei in the DC and VC of an adult brain are presumably embryonic *Poxn*-nuclei**. The nuclei of the *Poxn*-neurons in one hemisphere of an adult *w*; *CyO*/+; *Poxn-CD8*::*GFP UASrCD2/P{GawB}NP3503* brain, which is wild-type in *Poxn*, were numbered (Table 1), and their diameters measured and averaged over three orthogonal sections. The histograms for the dorsal (A) and ventral clusters (B) show the distributions of the nuclear size classes that are indicated in panels C and D for embryonic (red) and third instar larval (green) *Poxn*-nuclei. Consistent with the assumption that the largest nuclei correspond to those of embryonic *Poxn*-neurons, it was shown that the 20 largest *Poxn*-nuclei of the DC and the 12 largest *Poxn*-nuclei of the VC did not incorporate BrdU (Fig 1). The largest *Poxn*-nuclei have an average diameter of about 4.3 ± 0.23 μm (s.d.; 6 of the arbitrarily chosen units shown in the histograms correspond to 4.5 μm). **(C,D) Nuclear size distribution of *Poxn*-neurons along the anteroposterior axis in the adult brain**. The distributions of the size classes of *Poxn*-nuclei, shown in panels A (C) and B (D), are shown along the anteroposterior axis with respect to the Z-stack intervals indicated on the abscissa. A bias in the distribution of nuclear diameters of *Poxn*-neurons along the anteroposterior axis with larger nuclei closer to the posterior, as seen in late third instar larval brains (S7C and S7D Fig), is still observed in the DC (C) and VC (D).
(TIF)

S9 Fig Degenerate structure of ellipsoid body neuropil in adult brain of *Poxn* mutants. Frontal (**A-B’**) and horizontal (**C-D’**) 7 μm thick paraffin sections [116] of *w*^*1118*^ (A,C) and *w*^*1118*^; *Poxn*^*ΔM22-B5*^ *Poxn-SuperA* rescued (B,D) adult brains, and of *w*^*1118*^; *Poxn*^*ΔM22-B5*^ (A’), *w*^*1118*^; *Poxn*^*ΔM22-B5*^ *Poxn-Sbl-107* (B’,C’), and *w*^*1118*^; *Poxn*^*ΔM22-B5*^ *Poxn-Sbl-44* (D’) *Poxn* mutant adult brains. The EB and FB of mutant brains rescued by the *Poxn-SuperA* transgene (B,D) appear like the corresponding neuropils of wild-type brains (A,C). By contrast, the EB of *Poxn* mutants rescued by a *Poxn-Sbl* transgene (B’,C’,D’) resembles that of *Poxn* mutants (A’), which is degenerate, exhibiting a few globular structures in its place (arrowheads), whereas the FB appears as in wild-type brains. All panels show autofluorescence images recorded by wide-field microscopy with a 25x Plan-Neofluar objective lens, except panel B’ taken with a 40x Plan-Neofluar lens. EBC, ellipsoid body canal; NO, noduli. Scale bars: 25 μm.
(TIF)

S10 Fig Overlap with several GAL4 enhancer trap lines of Poxn expression in the ventral cluster of adult brains. **(A–C)** Colocalization (white) in nuclei of Poxn (green) and mCD8::GFP (A) or nuclear β-galactosidase (B,C) (red) expressed from targets of Gal4 enhancer trap lines, visualized by immunofluorescent staining, is shown in adult brain hemispheres of the genotypes *w*^*1118*^*/Y*; *P{GawB}GH146*/*P{w*^+^, *UAS-mCD8*::*GFP}* (A), *w*^*1118*^*/Y*; *P{w*^+^, *UAS-LacZ(nls)}*/+; *P{GawB}MZ699*/+ (B), and *w*^*1118*^ *P{GawB}KL107/Y*; *P{w*^+^, *UAS-LacZ (nls)}*/+ (C). Costaining of all *Poxn*-nuclei was corroborated by visual inspection in single layers of the Z-stacks. Panels show maximum intensity projections of CLSM sections of Z-stacks extending over 21 μm (A), 31 μm (B), and 33 μm (C) at 40x magnification. The scale bar is 20 μm and the same for all panels.
(TIF)
