# Supplementary figures and images for: Crucial roles of Pox neuro in the developing ellipsoid body and antennal lobes of the Drosophila brain
Source: PLoS One. 2017 Apr 25;12(4):e0176002. doi: 10.1371/journal.pone.0176002 (PMC5404782; doi:10.1371/journal.pone.0176002)

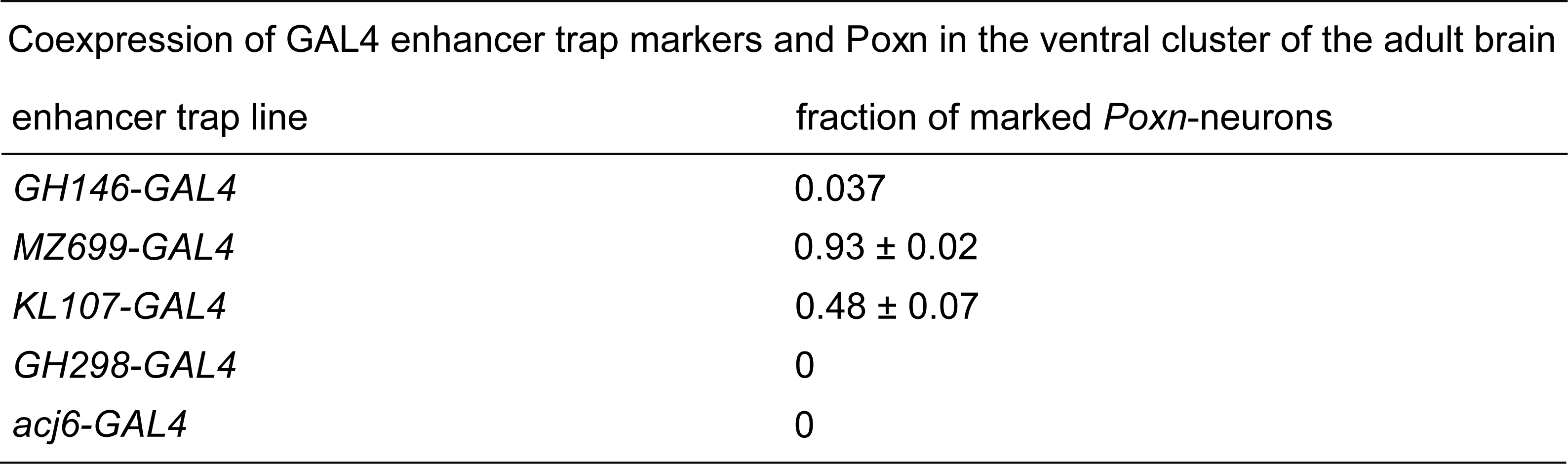

Supplement: S1 Table — The fractions of Poxn-neurons expressing the GAL4 enhancer trap lines in the VC of the adult brain were determined by examining their coexpression with the enhancer trap lines in single layers of CLSM sections, examples of which are shown as maximum intensity projections in S10 Fig. Standard deviations were determined from four ventral clusters each. The overlap of Poxn expression with that of the enhancer trap line GH146-GAL4 was constant. There was no coexpression of Poxn with GH298-GAL4 and acj6-GAL4. (TIF) [file pone.0176002.s001.tif]

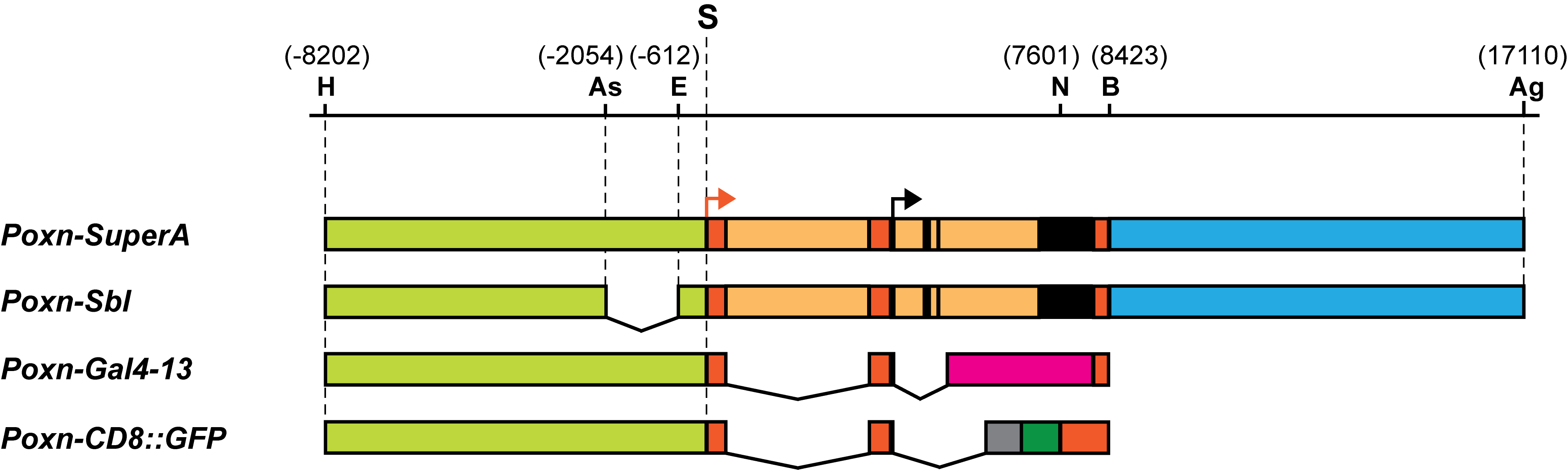

Supplement: S1 Fig — Maps of the Poxn constructs that rescue as transgenes all Poxn functions, Poxn-SuperA, or all but the Poxn brain function, Poxn-Sbl, and of the Poxn-driver, Poxn-Gal4-13, and Poxn-reporter construct, Poxn-CD8::GFP, are shown with regard to a restriction map of the Poxn locus (numbers in parentheses refer to distances in base pairs from the ‘upstream’ transcriptional start site S; cf. [19]). Poxn-Gal4-13 [19] and Poxn-CD8::GFP express Gal4 and the CD8::GFP fusion protein, respectively, under the control of the Poxn upstream enhancers. The composition of the Poxn constructs is illustrated in colors representing the Poxn upstream region (light green), 5’- and 3’-UTRs and out-of-frame Poxn coding region (dark orange), introns (light orange), coding region (black), and downstream region (light blue), and the coding regions of Gal4 (magenta), CD8 (gray), and GFP (dark green). The orange and black arrows indicate start and direction of transcription and translation, respectively. Restriction sites: Ag, AgeI; As, AscI; B, BamHI; E, EcoRI; H, HindIII; N, NotI. (TIF) [file pone.0176002.s002.tif]

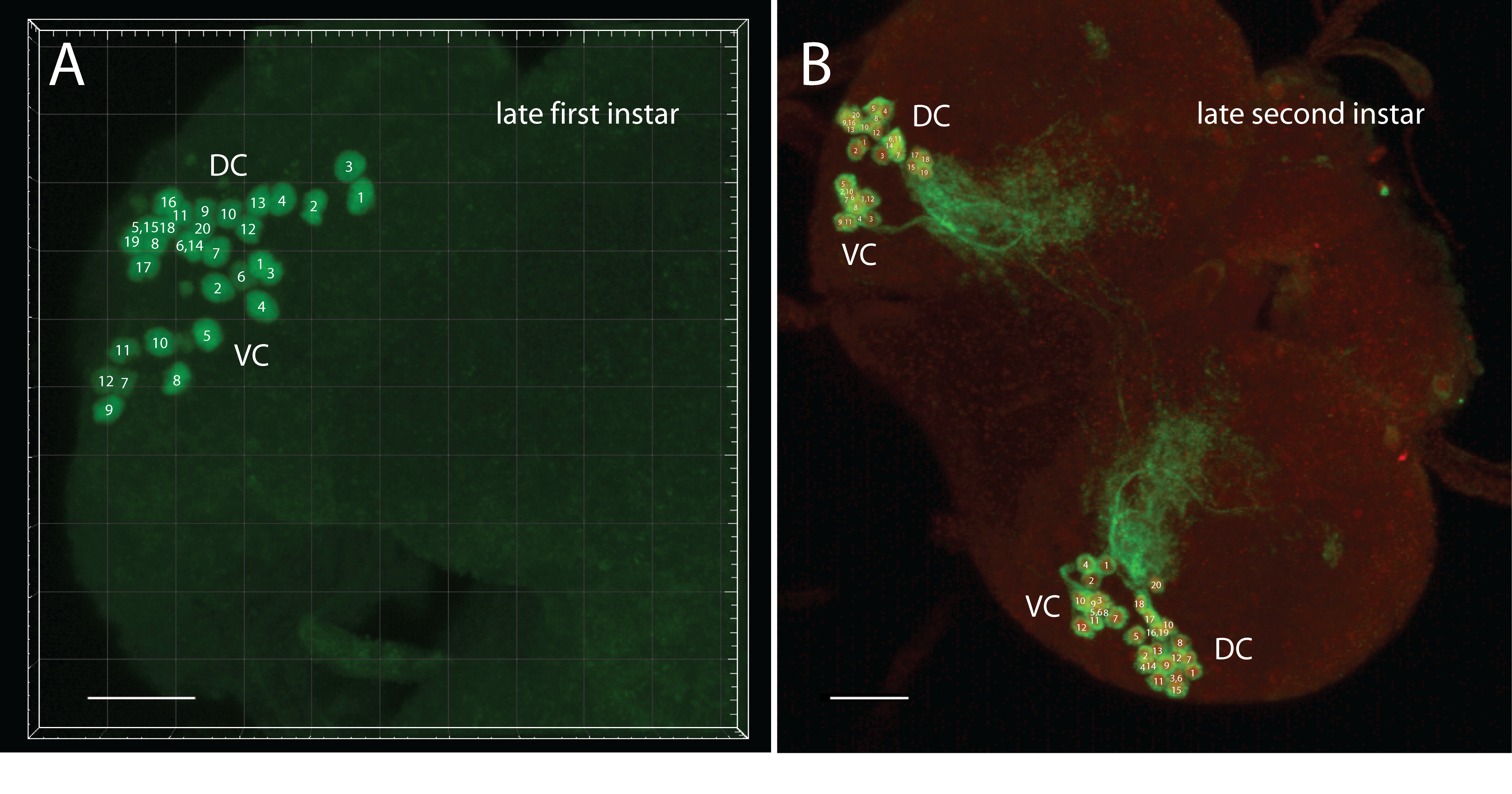

Supplement: S2 Fig — (A) The Poxn-nuclei in one hemisphere of an Ore-R late first instar larval brain (45 h AEL; Table 1), stained for Poxn (green) and Elav (red), are shown in an Imaris surpass view of the entire Z-stack extending over 43 μm at 63x magnification (same image as in Fig 2A). Only the green channel is shown. The Poxn-nuclei of the VC and DC are numbered from anterior to posterior, and all of these in the VC are anterior to those in the DC. Thus, Poxn-nuclei 14 and 15 of the DC are superimposed but posterior to Poxn-nuclei 6 and 5, respectively, as evident from inspection of individual layers of the Z-stack. Similarly, analysis of single layers showed that all Poxn-nuclei are positive for Elav, whereas some smaller green spots do not stain for Elav (not shown). (B) The Poxn-nuclei of a w1118; Poxn-CD8::GFP late second instar larval brain (68 h AEL; Table 1), stained for Poxn (red) and GFP (green), are shown as a maximum intensity projection of CLSM sections of a Z-stack extending over 50 μm at 20x magnification. Numbering of the cell bodies of Poxn-neurons in the DC and VC of the right and left hemispheres, shown in the upper and lower part of the image, is from anterior to posterior. The brain is the same as that shown in Fig 4B where only the green channel is shown. Scale bars: 10 μm (A) and 20 μm (B). (TIF) [file pone.0176002.s003.tif]

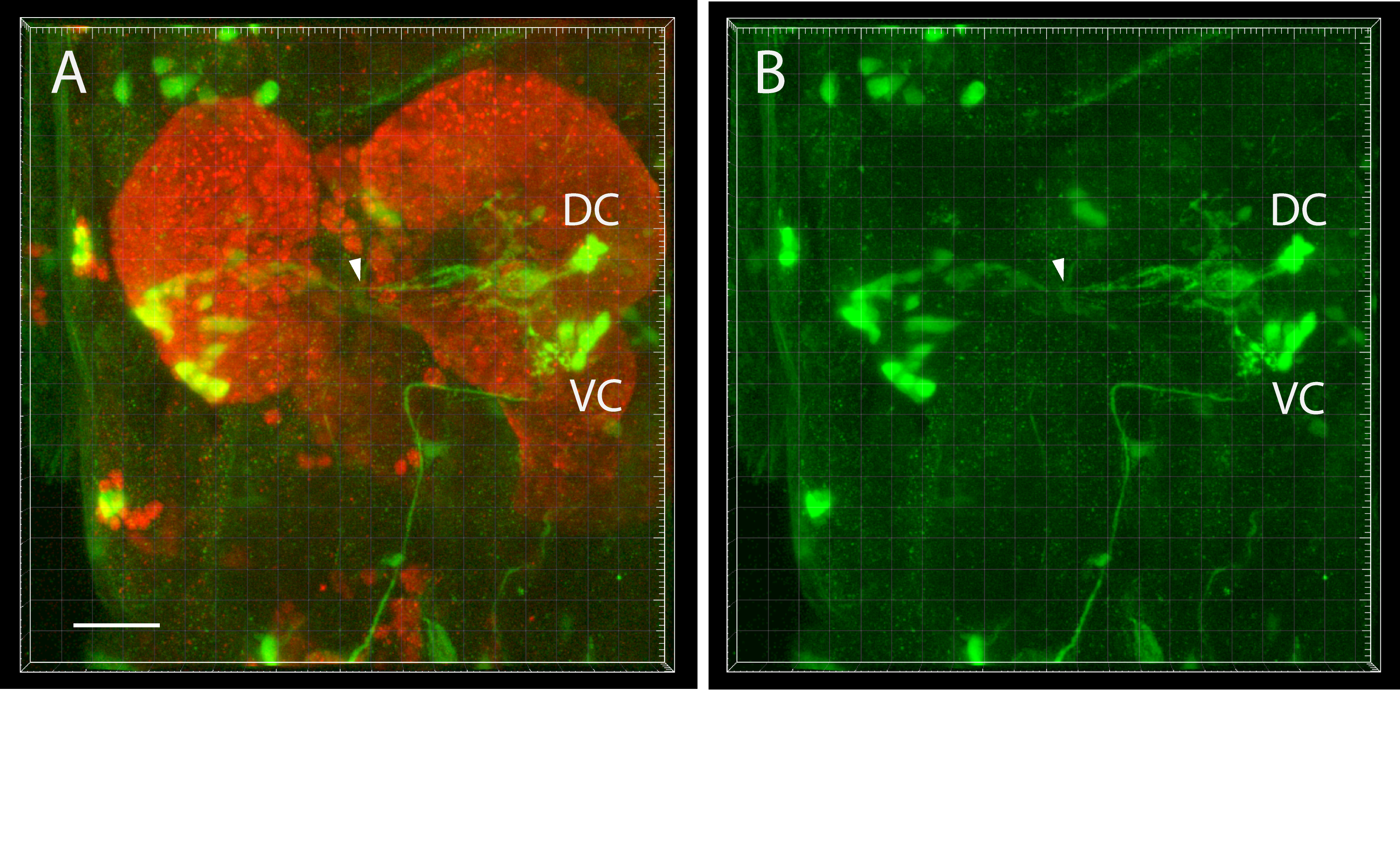

Supplement: S3 Fig — (A,B) Poxn-neurons in the brain of a w1118; Poxn-Gal4-13-1 UAS-GFP/TM6B stage 14 embryo are visualized by immunofluorescent staining for GFP (green) and Elav (red), and are analyzed in both channels (A) or only the green channel (B). Panels show an Imaris surpass view of a substack (that eliminates some cell bodies but shows all projections of Poxn-neurons) of a Z-stack extending over 42 μm at 63x magnification with anterior up. Inspection of single confocal layers reveals that all Poxn-expressing cells in the brain also express Elav and hence are post-mitotic neurons. While the Poxn-neurons of the VC do not yet show overt signs of neurite differentiation, the Poxn-neurons of the DC clearly differentiate neurites and extend axons along several tracts that appear to meet at the midline in the SEC (white arrowheads). Scale bar: 20 μm. (TIF) [file pone.0176002.s004.tif]

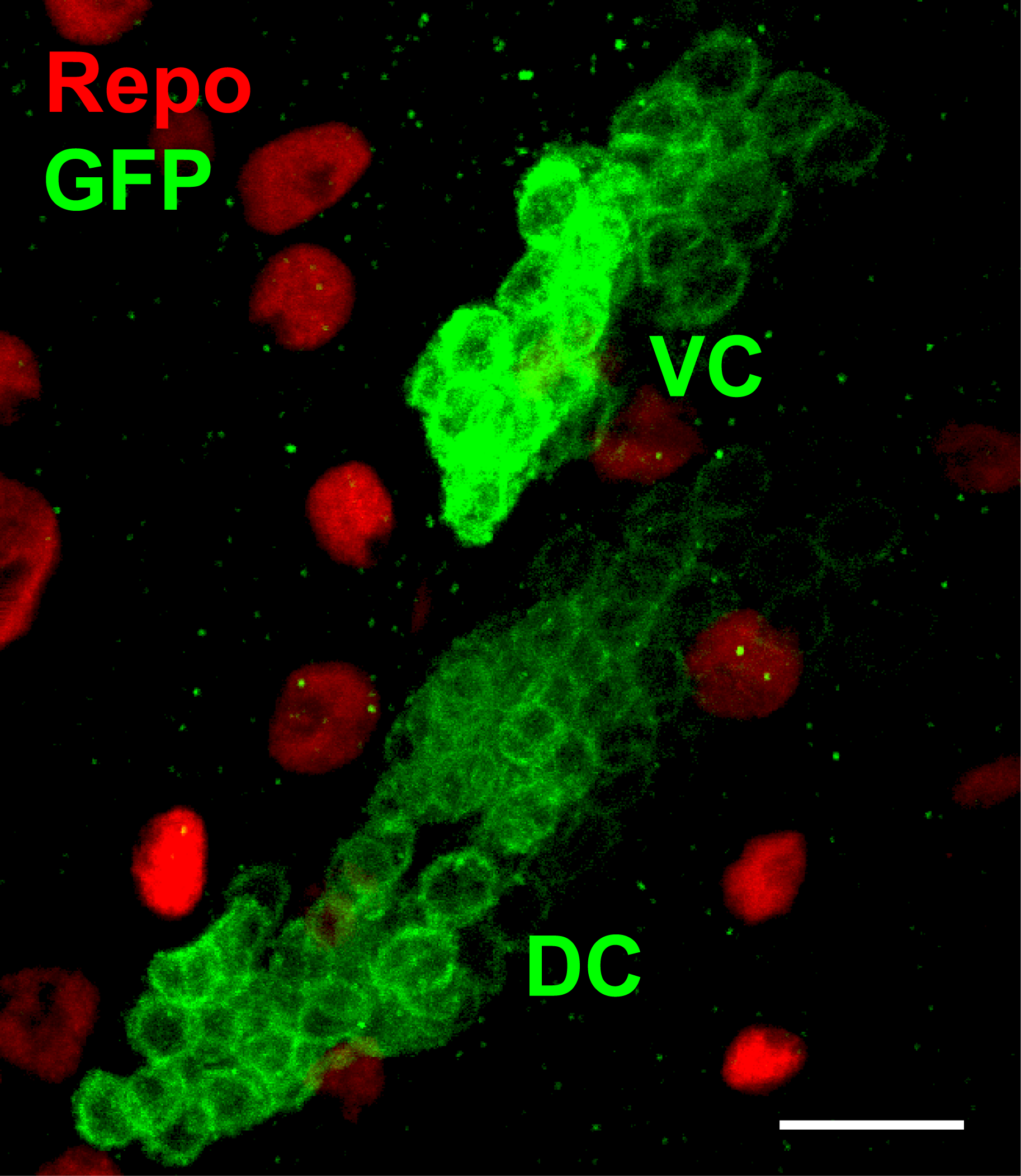

Supplement: S4 Fig — Cells expressing Poxn (green) and Repo (red) are visualized by immunofluorescent staining of the brain of a w1118; Poxn-CD8::GFP late third instar larva. The brain region shows a DC and VC as maximum intensity projection (of a Z-stack extending over 35 μm) along the A/P axis at 63x magnification. No colocalization of GFP and Repo is observed, which would be revealed as white pixels. Neurites are not visible because the image was taken at low exposure for optimal visibility of cell bodies. Scale bar: 10 μm. (TIF) [file pone.0176002.s005.tif]

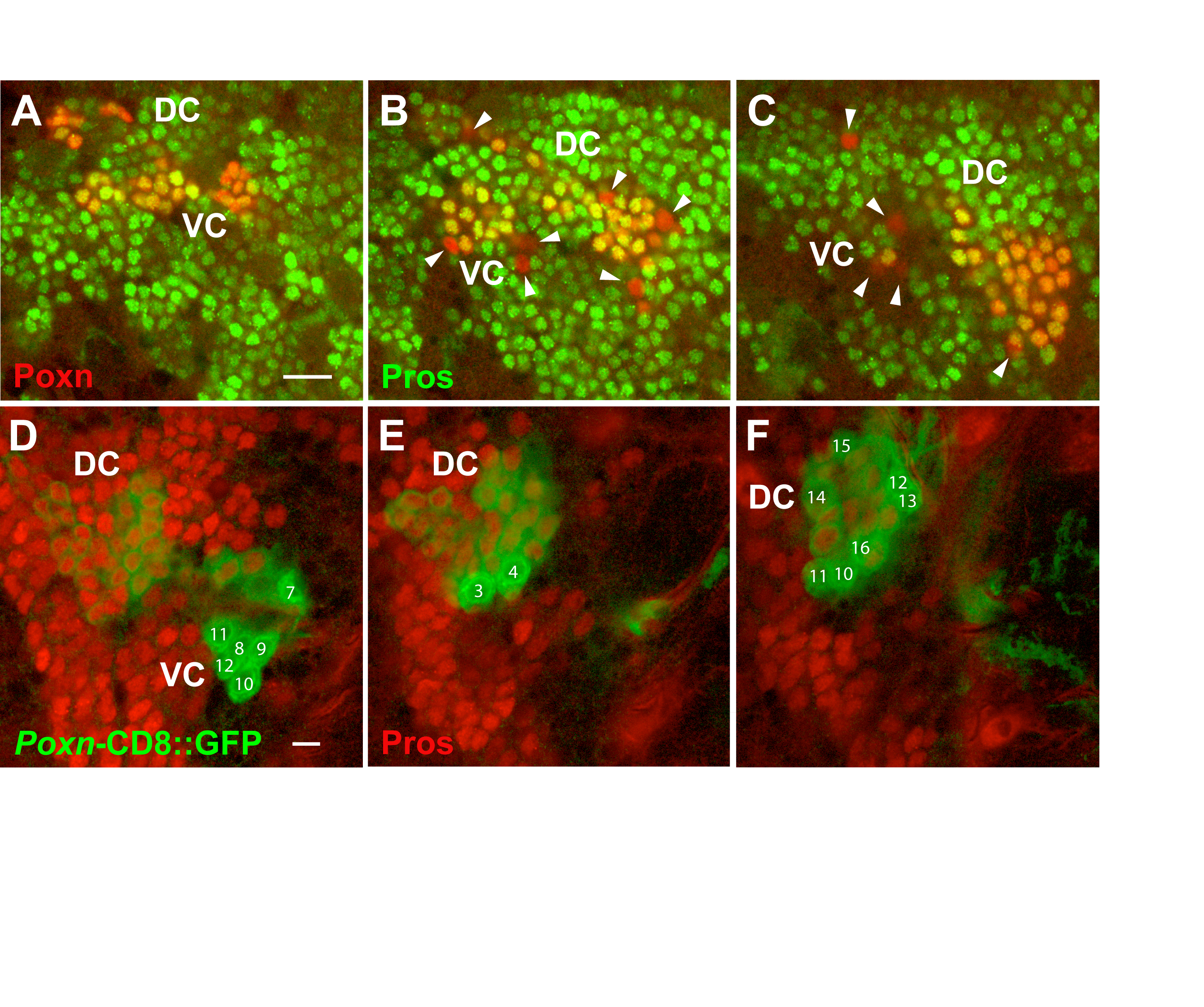

Supplement: S5 Fig — (A–C) The Poxn-nuclei in one hemisphere of an Ore-R late third instar larval brain, stained for Poxn (red) and Pros (green), are shown as 1 μm sections at 9–10 μm (A), 15–16 μm (B), and 21–22 μm (C) of a confocal Z-stack extending from 0 (anterior) to 36 μm (posterior). Note that all Poxn-nuclei stain for Pros except 20 of the DC and 12 of the VC, some of which are visible in panels B (4 and 3 in DC and VC, respectively) and C (2 and 3 in DC and VC, respectively), as indicated by arrowheads. These nuclei, which do not stain for Pros, are at the large end of the size distribution of Poxn-nuclei (S7A and S7B Fig), show the by far highest levels of Poxn protein, and are the same as those that stain for Poxn in late embryos or in first and second instar larvae (S2 Fig and Table 1). (D–F) The Poxn-neurons in one hemisphere of a w1118; Poxn-CD8::GFP late third instar larval brain, stained for Pros (red) and GFP (green), are shown as 1 μm sections at 16–17 μm (D), 22–23 μm (E), and 27–28 μm (F) of a confocal Z-stack extending from 0 (anterior) to 50 μm (posterior). A maximum intensity projection of the entire Z-stack in the green channel is shown in S6 Fig to which the numbers of Poxn-neurons that do not express Pros refer. These reveal neurites, in contrast to Poxn-neurons that express Pros, which show no overt signs of differentiation. All images were taken at 63x magnification. Scale bars: 10 μm (A–C) and 5 μm (D–F). (TIF) [file pone.0176002.s006.tif]

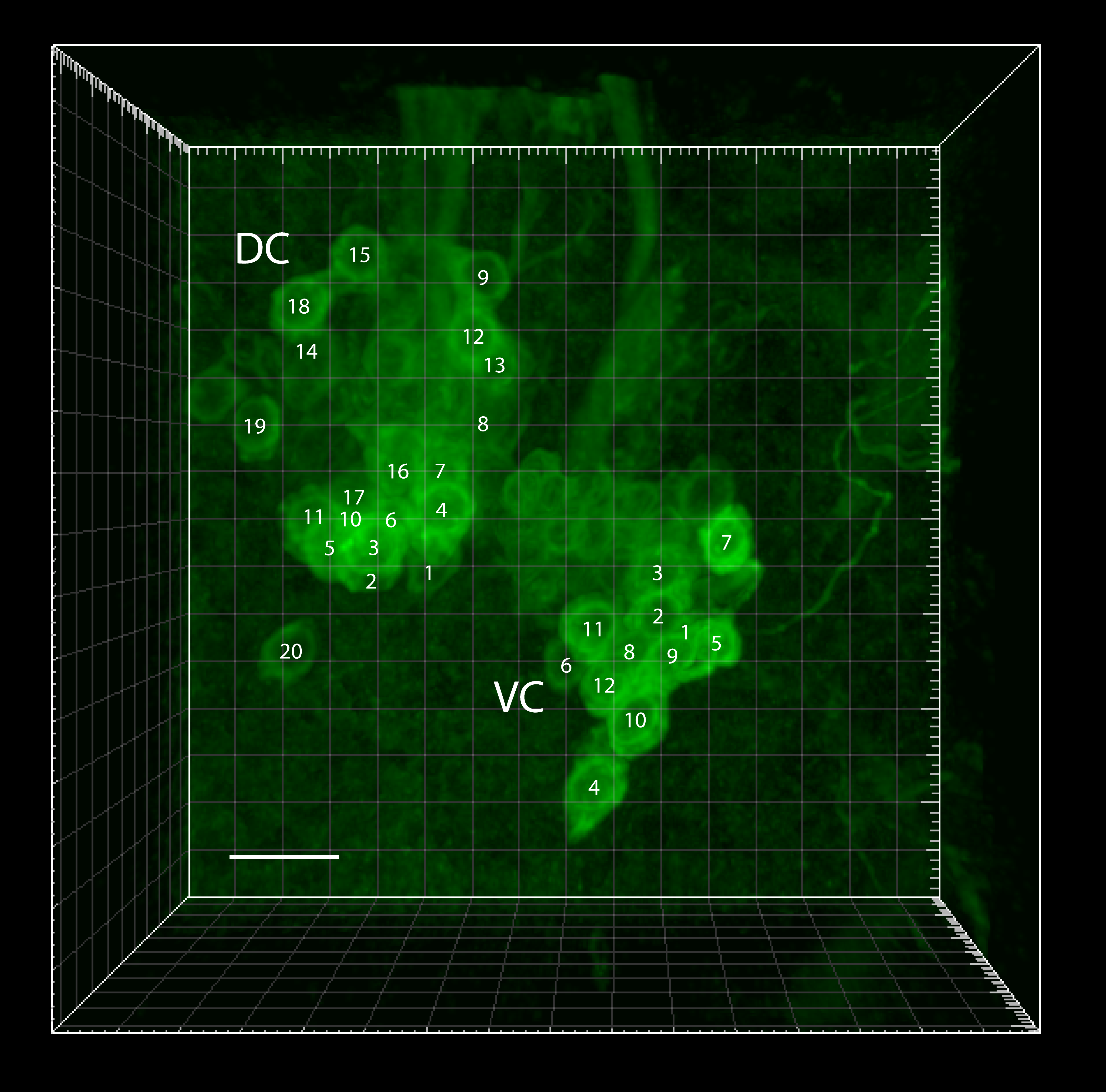

Supplement: S6 Fig — The Poxn-neurons in one hemisphere of a w1118; Poxn-CD8::GFP late third instar larval brain, stained for Pros (red; not shown) and GFP (green), are shown as an Imaris surpass view of the entire Z-stack extending over 51 μm at 63x magnification. Only the green channel is shown. The confocal picture is the same Z-stack of which three sections are shown in S5D–S5F Fig The Poxn-neurons in the DC and VC that do not express Pros are numbered from anterior to posterior (or ventral to dorsal of the flattened CNS), and all of these in the VC are anterior to those in the DC. Their numbers, 20 in the DC and 12 in the VC, correspond to those of the embryonic Poxn-neurons present by late embryogenesis and in first and second instar larvae (Table 1; S2 Fig). These embryonic Poxn-neurons of late third instar larvae also exhibit the highest levels of CD8::GFP, which is consistent with the highest levels of Poxn protein observed in the nuclei of these Poxn-neurons (S5A–S5C Fig). Scale bar: 10 μm. (TIF) [file pone.0176002.s007.tif]

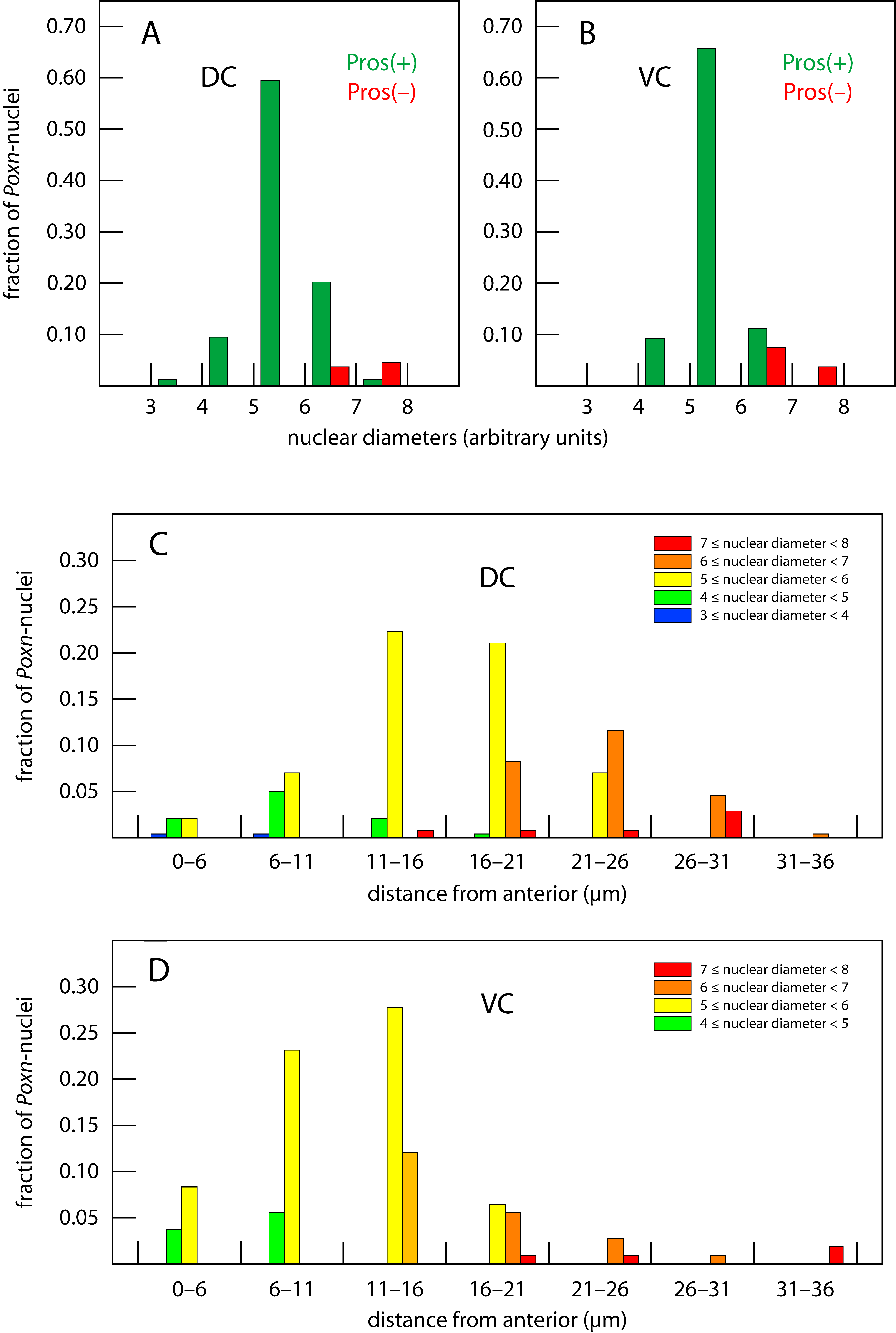

Supplement: S7 Fig — (A,B) Embryonic Poxn-neurons of late third instar larvae differ from larval Poxn-neurons by the absence of Pros and their larger nuclear size. The nuclei of the Poxn-neurons in one hemisphere of a late third instar Ore-R brain, three sections of which are shown in S5A–S5C Fig, were numbered (Table 1), and their diameters measured and averaged over three orthogonal sections. The histograms for the dorsal (A) and ventral clusters (B) show the distributions of the nuclear size classes that are indicated in panels C and D for Poxn-nuclei labeled (green) and not labeled by Pros (red). Note that there are 20 (DC) and 12 (VC) Poxn-nuclei that lack Pros, which equals the number of embryonic Poxn-neurons (Table 1), and that these have the largest nuclei. The largest Poxn-neurons have an average nuclear diameter of 3.4 ± 0.15 μm (s.d.; 7 of the arbitrarily chosen units shown in the histograms correspond to 3.5 μm). (C,D) Skewed nuclear size distribution of Poxn-neurons along the anteroposterior axis in the late third instar brain. The distributions of the size classes of Poxn-nuclei, shown in panels A (C) and B (D), are shown along the anteroposterior axis with respect to the Z-stack intervals indicated on the abscissa. In both the DC (C) and VC (D), there is a striking bias in the nuclear size distribution with larger nuclear diameters closer to the posterior. When plotted, the average nuclear diameters increase roughly in a linear fashion with increasing distance from the anterior. No distinction was made between nuclei displaying high, medium, and low levels of Pros or no Pros, classes that can be easily distinguished in sections (S5A–S5C Fig). (TIF) [file pone.0176002.s008.tif]

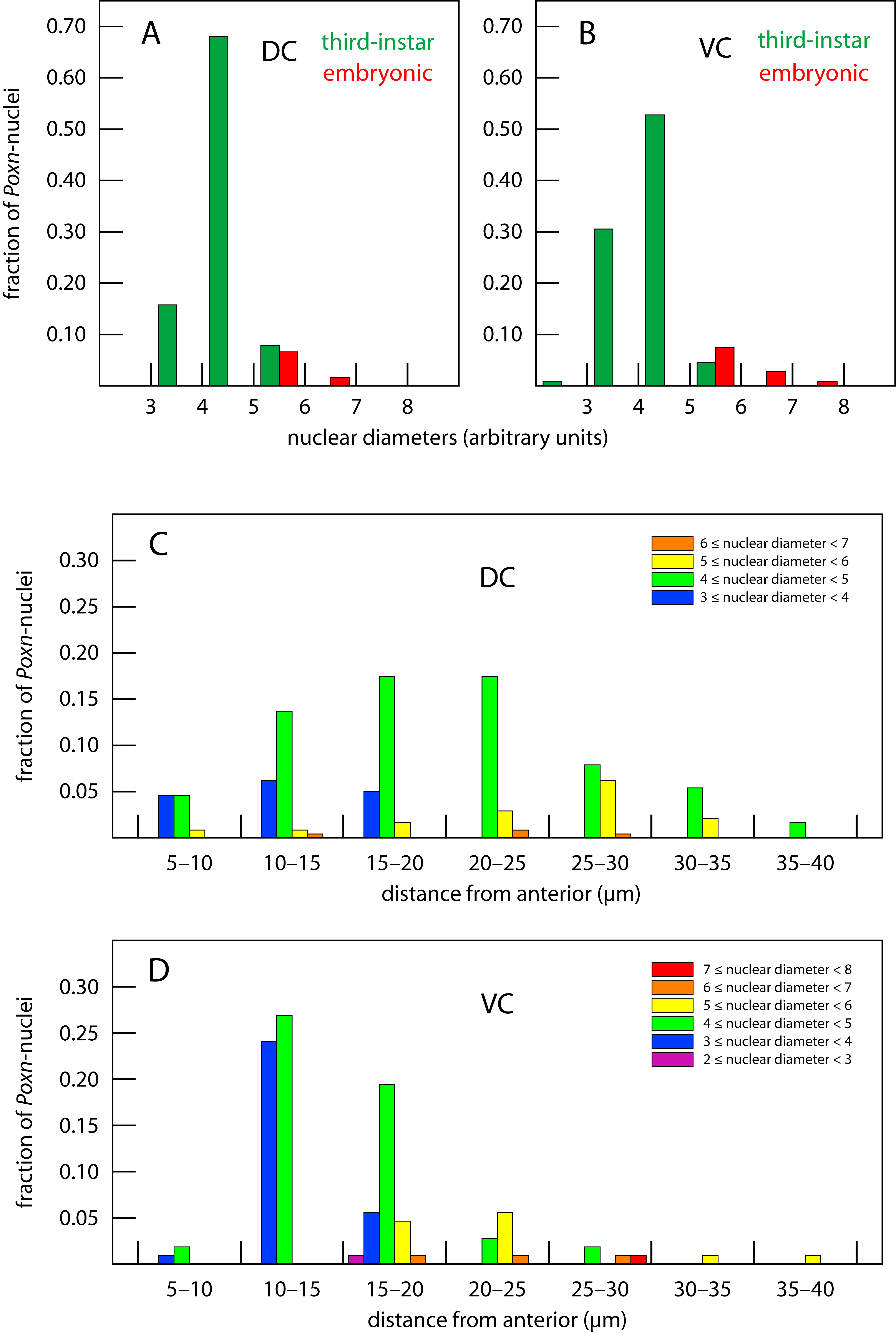

Supplement: S8 Fig — (A,B) The largest Poxn-nuclei in the DC and VC of an adult brain are presumably embryonic Poxn-nuclei. The nuclei of the Poxn-neurons in one hemisphere of an adult w; CyO/+; Poxn-CD8::GFP UASrCD2/P{GawB}NP3503 brain, which is wild-type in Poxn, were numbered (Table 1), and their diameters measured and averaged over three orthogonal sections. The histograms for the dorsal (A) and ventral clusters (B) show the distributions of the nuclear size classes that are indicated in panels C and D for embryonic (red) and third instar larval (green) Poxn-nuclei. Consistent with the assumption that the largest nuclei correspond to those of embryonic Poxn-neurons, it was shown that the 20 largest Poxn-nuclei of the DC and the 12 largest Poxn-nuclei of the VC did not incorporate BrdU (Fig 1). The largest Poxn-nuclei have an average diameter of about 4.3 ± 0.23 μm (s.d.; 6 of the arbitrarily chosen units shown in the histograms correspond to 4.5 μm). (C,D) Nuclear size distribution of Poxn-neurons along the anteroposterior axis in the adult brain. The distributions of the size classes of Poxn-nuclei, shown in panels A (C) and B (D), are shown along the anteroposterior axis with respect to the Z-stack intervals indicated on the abscissa. A bias in the distribution of nuclear diameters of Poxn-neurons along the anteroposterior axis with larger nuclei closer to the posterior, as seen in late third instar larval brains (S7C and S7D Fig), is still observed in the DC (C) and VC (D). (TIF) [file pone.0176002.s009.tif]

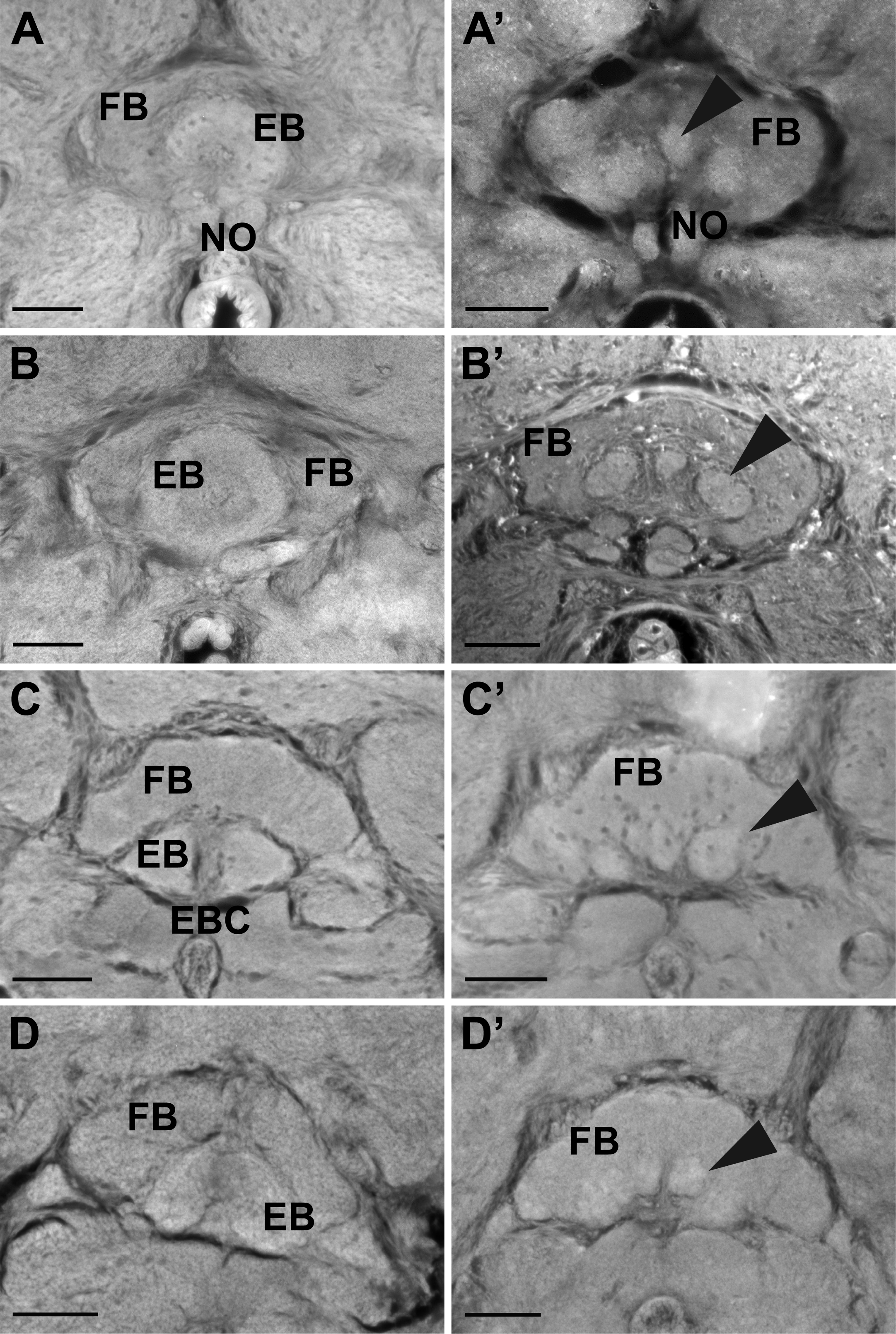

Supplement: S9 Fig — Frontal (A-B’) and horizontal (C-D’) 7 μm thick paraffin sections [116] of w1118 (A,C) and w1118; PoxnΔM22-B5 Poxn-SuperA rescued (B,D) adult brains, and of w1118; PoxnΔM22-B5 (A’), w1118; PoxnΔM22-B5 Poxn-Sbl-107 (B’,C’), and w1118; PoxnΔM22-B5 Poxn-Sbl-44 (D’) Poxn mutant adult brains. The EB and FB of mutant brains rescued by the Poxn-SuperA transgene (B,D) appear like the corresponding neuropils of wild-type brains (A,C). By contrast, the EB of Poxn mutants rescued by a Poxn-Sbl transgene (B’,C’,D’) resembles that of Poxn mutants (A’), which is degenerate, exhibiting a few globular structures in its place (arrowheads), whereas the FB appears as in wild-type brains. All panels show autofluorescence images recorded by wide-field microscopy with a 25x Plan-Neofluar objective lens, except panel B’ taken with a 40x Plan-Neofluar lens. EBC, ellipsoid body canal; NO, noduli. Scale bars: 25 μm. (TIF) [file pone.0176002.s010.tif]

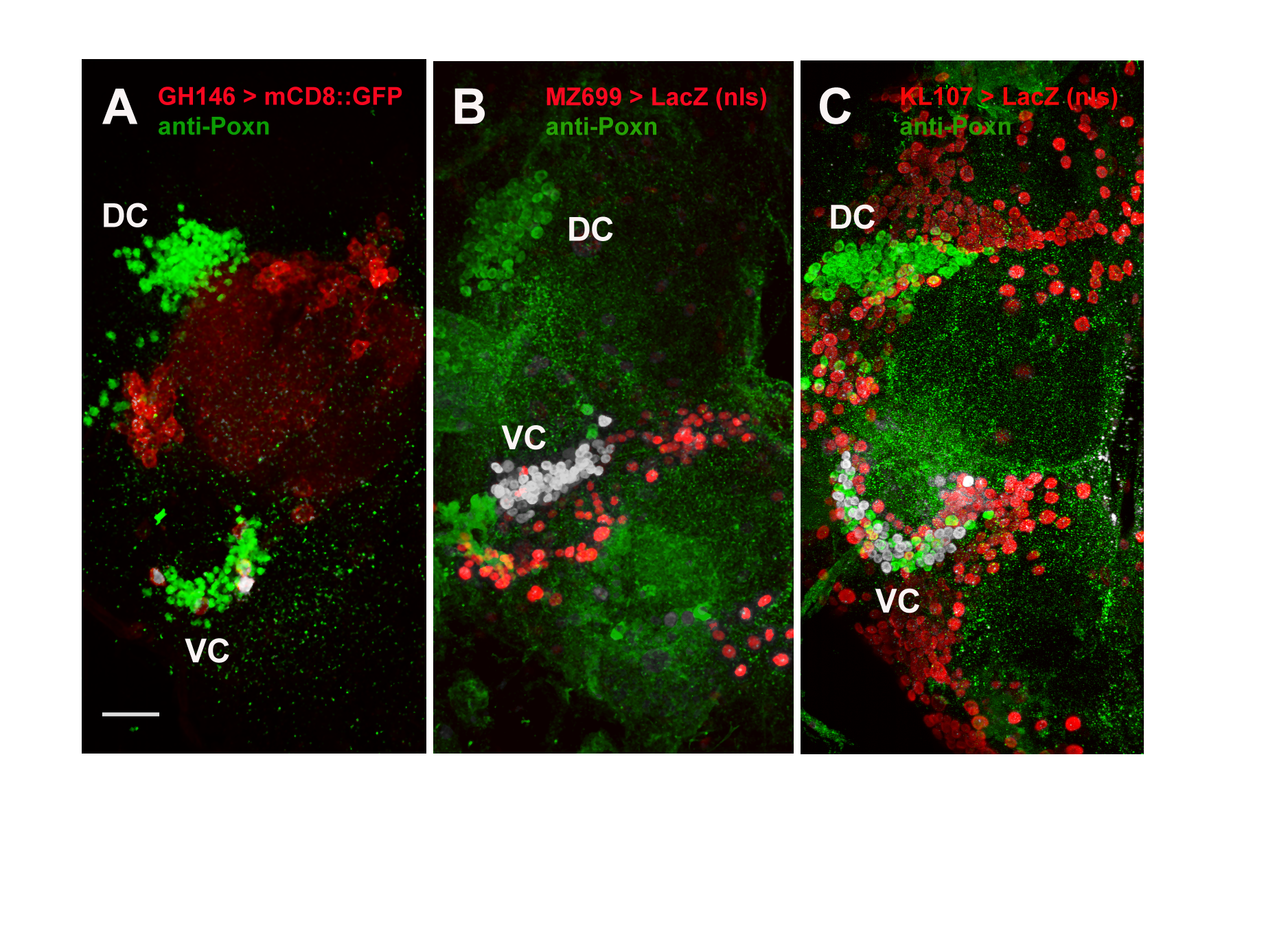

Supplement: S10 Fig — (A–C) Colocalization (white) in nuclei of Poxn (green) and mCD8::GFP (A) or nuclear β-galactosidase (B,C) (red) expressed from targets of Gal4 enhancer trap lines, visualized by immunofluorescent staining, is shown in adult brain hemispheres of the genotypes w1118/Y; P{GawB}GH146/P{w+, UAS-mCD8::GFP} (A), w1118/Y; P{w+, UAS-LacZ(nls)}/+; P{GawB}MZ699/+ (B), and w1118 P{GawB}KL107/Y; P{w+, UAS-LacZ (nls)}/+ (C). Costaining of all Poxn-nuclei was corroborated by visual inspection in single layers of the Z-stacks. Panels show maximum intensity projections of CLSM sections of Z-stacks extending over 21 μm (A), 31 μm (B), and 33 μm (C) at 40x magnification. The scale bar is 20 μm and the same for all panels. (TIF) [file pone.0176002.s011.tif]
